# A TNFR1–UBCH10 axis drives lung squamous cell carcinoma dedifferentiation and metastasis through a cell-autonomous signaling loop

**DOI:** 10.1038/s41419-022-05308-4

**Published:** 2022-10-21

**Authors:** Zuoxiang Xiao, Gongping Shi, Sichuan Xi, Amit Kumar Singh, Jami Willette-Brown, Xin Li, Feng Zhu, Ling Su, Xiaolin Wu, David S. Schrump, Yinling Hu

**Affiliations:** 1 Cancer Innovation Laboratory, Center for Cancer Research, National Cancer Institute, National Institutes of Health, Frederick, MD 21702 USA; 2 Thoracic Surgery Branch, Center for Cancer Research, National Cancer Institute, Bethesda, MD 20892 USA; 3 Genomics Technology Laboratory, Frederick National Laboratory for Cancer Research, Frederick, MD 21701 USA

**Keywords:** Cancer models, Cancer

## Abstract

Tumor necrosis factor receptor 1 (TNFR1), encoded by *TNFRSF1A*, is a critical transducer of inflammatory pathways, but its physiological role in human cancer is not completely understood. Here, we observed high expression of TNFR1 in many human lung squamous cell carcinoma (SCCs) samples and in spontaneous lung SCCs derived from kinase-dead *Ikkα* knock-in (*KA/KA*) mice. Knocking out *Tnfrf1a* in *KA/KA* mice blocked lung SCC formation. When injected via tail vein, KAL^LU+^ lung SCC cells that highly expressed TNFR1/TNF, Sox2, c-Myc, Twist1, Bcl2, and UBCH10, generated dedifferentiated spindle cell carcinomas with epithelial–mesenchymal transition markers in mouse lungs. In contrast, KAL^LU+^ cells with silenced TNFR1 and KAL^LU-^ cells that expressed low levels of TNFR1 generated well-differentiated lung SCCs and were less tumorigenic and metastatic. We identified a downstream effector of TNFR1: oncogenic UBCH10, an E2 ubiquitin-conjugating enzyme with targets including Twist1, c-Myc, and Sox2, which enhanced SCC cell dedifferentiation. Furthermore, Tg-K5.TNFR1;*KA/KA* mice, which expressed transgenic TNFR1 in keratin 5-positve epithelial cells, developed more poorly differentiated and metastatic lung SCCs than those found in *KA/KA* mice. These findings demonstrate that an overexpressed TNFR1–UBCH10 axis advances lung carcinogenesis and metastasis through a dedifferentiation mechanism. Constituents in this pathway may contribute to the development of differentiation-related therapies for lung SCC.

## Introduction

Lung cancer confers the highest cancer mortality rate worldwide. Two major types of lung cancer, lung squamous cell carcinoma (SCC) and lung adenocarcinoma (ADC), are non-small cell lung cancer with distinct histological features and different genomic alterations [1, 2]. SCC is derived from keratin 5 (KRT5 or K5)-positive basal cells of the pseudostratified airway epithelium in the upper and central lungs, and ADC from the epithelial cells of alveoli [3, 4]. Lung SCC begins with basal epithelial stem cell hyperproliferation, then progresses to well-differentiated SCC, poorly differentiated SCC, and dedifferentiated spindle cell carcinoma, the final stage of SCC. Understanding SCC pathogenesis is essential to advancing clinical approaches to reduce mortality from these neoplasms.

Much existing knowledge of lung SCC development derives from animal models. *KrasLkb*^*L/L*^ mice specifically express the activated KRAS and serine kinase 11 (*Lkb1*) deletion and generate spontaneous lung SCC and mixed SCC and ADC [5]. Work in this model indicates the AKT, PTEN, and mTOR signaling cascade in SCC pathogenesis [6, 7]. Kinase-dead *Ikkα* knockin (*KA/KA*) mice develop spontaneous lung SCC characterized by well-differentiated keratin pearls and increased infiltration of pro-tumor macrophages [3, 8, 9]. Depletion of these macrophages prevents lung SCC development. The molecular mechanisms for lung SCC found in these mouse models may represent two distinct pathways.

Tumor necrosis factor receptor 1 (TNFR1), one member of the TNFR superfamily, is broadly expressed on the surface of various cell types, particularly epithelial cells. Chemical carcinogens, e.g., 7,12-dimethylbenz[a]anthracene and 12-0-tetradecanoylphorbol 13-acetate, induce *H-ras*-mutant skin papilloma and SCC [10, 11], but deletion of the *Tnfrsf1a* mouse gene encoding TNFR1 dampens chemical carcinogen-induced skin and liver carcinogenesis [12–16]. In contrast, overexpression of *Drosophila* TNFR promotes tumor invasiveness [17]. Yet a mechanistic role for TNFR1 in tumorigenesis is not defined.

Notably, TNFR1 stimulates a major inflammatory pathway, but whether TNFR1 plays a cell-autonomous role in oncogenesis is unknown. Here, we detected increased levels of TNFR1 mRNA and protein in many human lung SCCs and lung SCCs obtained from *KA/KA* mice. Deleting *Tnfrsf1a* prevented lung SCC development in these mice. We analyzed the ability of lung SCC cell lines with differing TNFR1 expression to form tumors and generated TNFR1 transgenic mice. Overexpressing TNFR1 resulted in dedifferentiated and metastatic spindle cell carcinomas. In contrast, silencing TNFR1 reduced levels of stemness, Twist1 (an epithelial–mesenchymal transition–EMT), and oncogenic UBCH10, an E2 ubiquitin-conjugating enzyme [18, 19], converted dedifferentiated spindle cell carcinoma to well-differentiated SCC, and inhibited metastasizing SCCs. We identified UBCH10 as a functional target of TNFR1 for promoting SCC dedifferentiation. This unique TNFR1–UBCH10 signaling axis represents a potential target for differentiation-related therapy for lung SCC.

## Results

### Human lung SCCs exhibit elevated expression of TNFR1 and transgenic TNFR1 promotes dedifferentiated and metastatic lung SCC development in mice

**Fig. 1:**
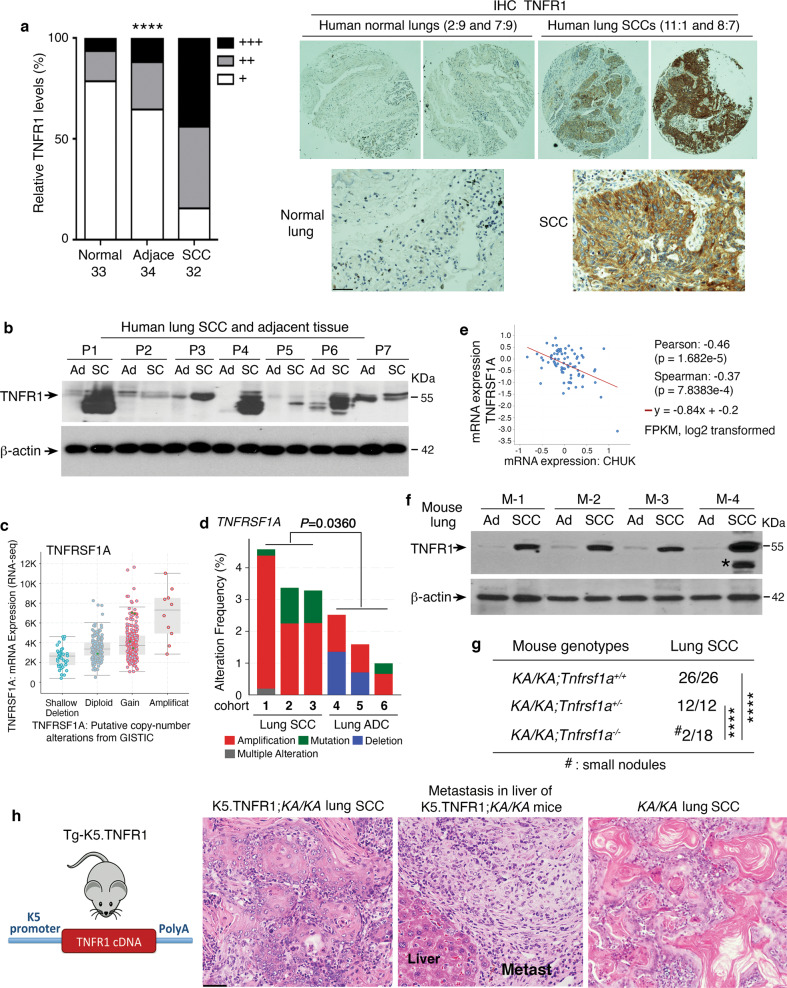
Overexpression of TNFRSF1A in human and mouse lung SCC. **a** Left: comparing the intensities of immunohistochemical (IHC) staining with an anti-TNFR1 antibody in 33 normal, 34 adjacent, and 32 lung SCC samples in an array (BC04118, Biomax). +++, strong staining; ++, moderate staining; +, weak staining. Comparison among these groups; ****P < 0.0001; Chi-squared test. Right: representative images of TNFR1 IHC staining in normal human lungs and lung SCCs. Numbers on the top of the images represent the location of the samples in the array (Fig. S1a). Scale bar: 50 μM. **b** TNFR1 protein levels detected by immunoblotting of human lung SCCs (SC) and in adjacent tissue samples (Ad). P, patient; β-actin, a protein-loading control. **c** TNFRSF1A expression levels, as detected by using RNA sequencing (RNA-seq), in a human lung SCC cohort (TCGA, PanCancer Atlas, *n* = 466, cBioPortal). **d** *TNFRSF1A* DNA alterations in three human lung SCC cohorts (1: TCGA, Firehose Legacy; 2: TCGA, Nature 2012; 3: TCGA, PanCancer Atlas, cBioPortal) and in three human lung ADC cohorts (1: TCGA, Firehose Legacy; 2: TCGA PanCancer Atlas; and 3: OncoSG, Nat Genet 2020, cBioPortal). Mean ± SEM (three repeats); Student’s *t*-test. **e** Co-expression of CHUK (IKKα) and TNFRSF1A in a human lung SCC cohort obtained from Cell 2021, CPTAC: Clinical Proteomic Tumor Consortium, (cBioPortal for Cancer Genomics). FPKM, Fragments Per Kilobase of transcript per Million mapped reads. *P*-values are indicated in this panel. **f** TNFR1 protein levels detected by immunoblotting in mouse lung SCCs isolated from *KA/KA* mice and in adjacent tissues. M, mouse; *, indicating a band that may represent an oligomer, a nonspecific band, or a cleaved TNFR1; β-actin, a protein-loading control. **g** Incidence of lung SCCs in *KA/KA;Tnfrsf1a*^*+/+*^, *KA/KA;Tnfrsf1a*^*+/-*^, and *KA/KA;Tnfrsf1a*^*-/-*^ mice. Numbers indicate mouse numbers. ****P < 0.0001; Chi square test. **h** Left: Generation of transgenic Tg-K5.TNFR1 mice. K5, keratin 5; Tg, transgenic. Right: Representative H&E-stained images of K5.TNFR1;*KA/KA* lung SCC and metastasis (Metast) to the liver, and of *KA/KA* lung SCC. Scale bar: 40 μM.

To assess relevance of TNFR1 expression levels to human lung SCC development, we used immunohistochemical (IHC) staining to examine TNFR1 levels in a tissue array containing 32 human lung SCCs, 34 adjacent tissues, and 33 normal lung sections. TNFR1 staining was significantly greater in lung SCC samples compared to adjacent tissues and normal lungs (Fig. 1a, left and right, and Fig. S1a). Consistent with this, immunoblotting and RT-PCR detected higher TNFR1 levels in human SCCs than in adjacent tissues (Fig. 1b and Fig. S1b). We also analyzed expression of *TNFRSF1A*, the gene encoding TNFR1, in 466 human lung SCCs from The Cancer Genome Atlas (TCGA) database (PanCancer Atlas, cBioportal); gene expression was elevated in a large population of samples (Fig. 1c). Many human lung ADCs (TCGA, PanCancer Atlas, *n* = 507) also had increased *TNFRSF1A* expression (Fig. S1c). Moreover, we compared *TNFRSF1A* genomic alterations in three human lung SCC and three human lung ADC cohorts and found that *TNFRSF1A* amplification was a major type of genomic alteration in the SCC cohorts compared to ADC (Fig. 1d). Whereases a large proportion of human lung SCC samples (TCGA, PanCancer Atlas) expressed reduced *CHUK* that encodes IKKα (Fig. S1d, left). Consistently, IKKα amounts were inversely correlated with TNFRSF1A expression in human lung SCC samples (Fig. 1e and Fig. S1d, right). Thus, the expression patterns of IKKα and TNFR1 are reciprocal. Together, these analyses identified increased TNFRSF1A/TNFR1 mRNA and protein levels in many human lung SCCs.

We used immunoblotting to examine TNFR1 levels in spontaneous lung SCCs isolated from *KA/KA* mice [3] and detected higher TNFR1 levels in SCCs than in adjacent tissues (Fig. 1f). To study the function of TNFR1 in lung SCC development, we generated *KA/KA*;*Tnfrsf1a*^*-/-*^ mice on an FVB background and found that *Tnfrsf1a* ablation inhibited lung SCC development (Fig. 1g and Fig. S1e). *KA/KA*;*Tnfrsf1a*^*+/-*^ mice still developed lung SCC, which is likely due to lung inflammation [3]. On the other hand, we generated transgenic Tg-K5.TNFR1 mice on an FVB background that overexpress TNFR1 in K5-positive lung epithelial cells [20] and these mice did not show lung phenotypes (Fig. 1h, left, and Fig. S1f, left and right). Tg-K5.TNFR1;*KA/KA* mice died earlier than *KA/KA* mice because of enhanced autoinflammatory phenotypes [21, 22]. Male and female *KA/KA* mice aged 6–10 months develop lung SCC and die [3]. Importantly, lung SCCs were already present in some of these Tg-K5.TNFR1;*KA/KA* mice at between 3 and 6 months of age, earlier than in *KA/KA* mice. These K5.TNFR1;*KA/KA* lung SCCs were poorly differentiated, lacked keratin pearls, and showed metastasis to the liver; whereas *KA/KA* mice generated well-differentiated lung SCC with keratin pearls, a terminal differentiation feature (Fig. 1h, right), indicating that elevated TNFR1 expression in K5-positive epithelial cells drives a dedifferentiation program in vivo, which leads to poorly differentiated and metastatic lung SCC development.

### TNFR1 supports dedifferentiated spindle cell carcinoma formation in mouse models

**Fig. 2:**
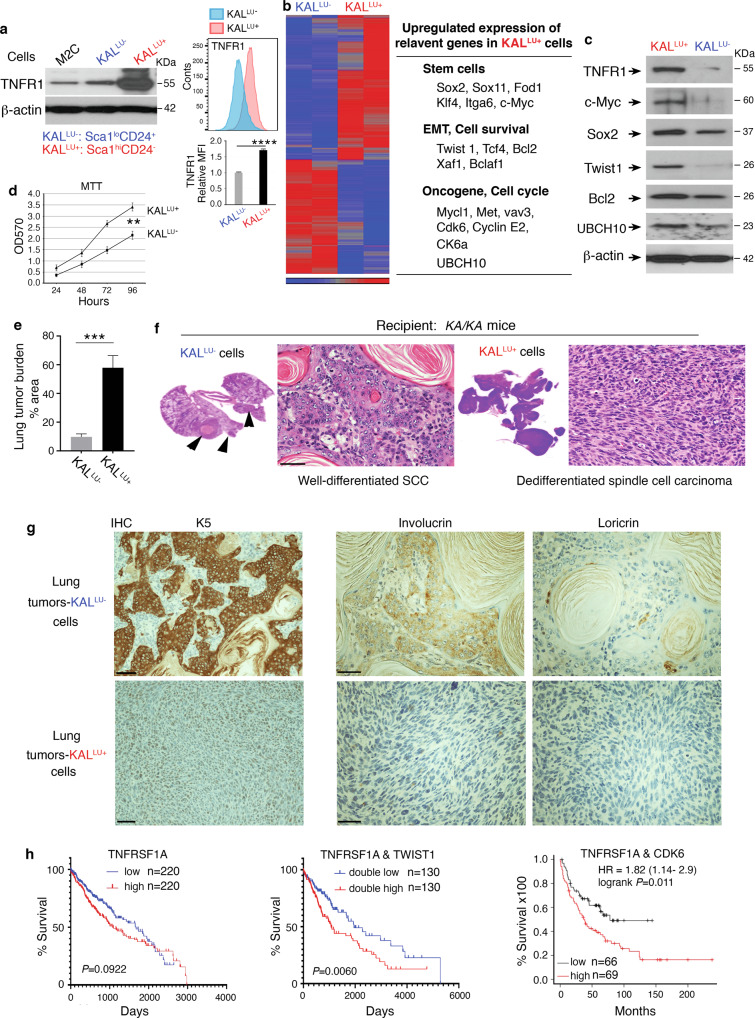
High-TNFR1 levels are associated with dedifferentiated lung spindle cell carcinoma. **a** Left: TNFR1 protein levels detected by immunoblotting in M2C, KAL^LU-^ (Scal^lo^CD24^+^CD44^+^) and KAL^LU+^ (Scal^hi^CD24^-^CD44^+^) cells. lo, low; hi, high; β-actin, a protein-loading control. Right: Flow cytometry analyzes TNFR1 expression levels (MFI, Mean Fluorescence Intensity) in KAL^LU-^ and KAL^LU+^ cells (high panel, images; low panel, statistical analysis for MFI of TNFR1 staining). Mean ± SD (three repeats) ****P < 0.0001; Student’s *t*-test. **b** Left: Heatmap comparing the gene expression profiles of KAL^LU-^ and KAL^LU+^ cells by gene array analyses. Right: Some upregulated genes in KAL^LU+^ cells compared to KAL^LU-^ cells. **c** Immunoblotting analysis verifying the expression of listed genes from Fig. 2b in KAL^LU-^ and KAL^LU+^ cells. β-actin, a protein-loading control. **d** Comparison of KAL^LU-^ and KAL^LU+^ cell proliferation using the MTT assay. Mean ± SEM (three repeats). **P < 0.01; Student’s *t*-test. **e** Tumor burden in the lungs generated by KAL^LU-^ and KAL^LU+^ cells in *KA/KA* mice (*n* = 5/group). Mean ± SD; ***P < 0.001; Student’s *t*-test. **f** Representative images of a well-differentiated lung SCC generated by KAL^LU-^ cells and spindle cell carcinoma generated by KAL^LU+^ cells in the lungs of *KA/KA* mice with the histological morphology stained with hematoxylin and eosin (H&E). Scale bar: 50 μM. **g** IHC staining of SCCs obtained from tail-vein injected KAL^LU-^ and KAL^LU+^ cells with anti-K5, anti-involucrin, and anti-loricrin antibodies. Scale bar: 40 μM. **h** Survival curves of patients with lung SCCs expressing high TNFRSF1A or low TNFRSF1A levels (left), double-high TNFRSF1A and TWIST1 or double-low TNFRSF1A and TWIST1 levels (middle); and double-high TNFRSF1A and CDK6 or low TNFRSF1A levels (right). These data were obtained from OncoLnc.

We previously established a KAL^LU^ cell line from *KA/KA* lung SCCs [3]. To investigate the cell-autonomous activity of TNFR1 for SCC cell differentiation, here, using cell surface markers of stemness, we generated KAL^LU-^ (Scal^lo^CD24^+^) and KAL^LU+^ (Scal^hi^CD24^-^) cell lines (Fig. 2a, left, and S2a). KAL^LU+^ cells expressed higher levels of TNFR1 than KAL^LU-^ cells and M2C cells, which are derived from murine lung epithelia [3] (Fig. 2a, left). Because TNFR1 is often present in large amounts in the Golgi and less on the cell surface [23, 24], we used flow cytometry to analyze TNFR1 expression and confirmed significantly higher levels of TNFR1 in KAL^LU+^ cells than in KAL^LU-^ cells (Fig. 2a, right-high and low panels), indicating increased TNFR1 expression on the surface of KAL^LU+^ cells. Furthermore, analysis of gene expression profiles revealed that KAL^LU+^ cells highly expressed genes that encode stemness markers (Sox2, Sox11, Itga6, and c-Myc), differentiation and survival molecules (Twist1, Tcf4, Bcl2, Xaf1, and Bclaf1), and oncogenes (Met, CDK6, Cyclin E2, and UBCH10), as compared to KAL^LU-^ cells (Fig. 2b). Elevated expression of TWIST1, which mediates epithelial–mesenchymal transition (EMT) with dedifferentiation features [25, 26], and UBCH10, which is encoded by *UBE2C* and is an E2 ubiquitin-conjugating enzyme, were detected in many human lung SCCs (Fig. S2b, left and middle). Overexpression of UBCH10 causes chromosome missegregation and results in multiple types of spontaneous tumors, including lung tumors in mice [18, 19] and it also upregulates the levels of c-Myc, TWIST1, and other oncogenes in human tumors [27–29]. Indeed, UBCH10 was highly expressed in lung SCC derived from *KA/KA* mice compared to WT lungs (Fig. S2b, right). Immunoblotting verified higher expression of TNFR1, c-Myc, Sox2, Twist1, and UBCH10 in KAL^LU+^ cells than in KAL^LU-^ cells (Fig. 2c). These molecular changes indicate that KAL^LU+^ cells are likely to be more oncogenic. Consistent with this, KAL^LU+^ cells grew faster than KAL^LU-^ cells (Fig. 2d).

We injected KAL^LU+^ (2 × 10^5^ cells per mouse) and KAL^LU-^ (1 × 10^6^ cells per mouse) cells into the tail vein of 6-week-old *KA/KA* mice. At 3 weeks after injection, the tumor burden in the lungs induced by KAL^LU+^ cells was significantly higher than that induced by KAL^LU-^ cells (Fig. 2e). KAL^LU-^ cells generated well-differentiated SCCs with keratin pearls, while KAL^LU+^ cells generated spindle cell carcinomas, a final-stage of SCC, in the lungs of *KA/KA* mice (Fig. 2f and Fig. S2c). Spontaneous lung SCC is found in 6–10-month-old *KA/KA* mice and is not detected in 2–3-month-old *KA/KA* mice [3]. Thus, these observed lung tumors were derived from injected KAL^LU+^ and KAL^LU-^ cells. IHC examination detected strong staining for the basal cell marker K5, positive staining for the intermediate differentiation marker involucrin, and weak staining for the terminal differentiation marker loricrin in KAL^LU-^ cell–generated well-differentiated SCCs (Fig. 2g). A low level of K5 was detected in KAL^LU+^ cell–generated spindle cell carcinomas but not involucrin or loricrin. These results suggest that the different expression levels of TNFR1 in SCC cells may decide their differentiation status.

Furthermore, analyses of human lung SCC cohorts from the TCGA database showed that patients with lung SCC expressing high levels of *TNFRSF1A* tended to have reduced survival compared to lung SCC patients expressing low levels of TNFRSF1A (Fig. 2h, left). Lung SCC patients expressing high levels of double *TNFRSF1A* and *TWIST1* showed significantly reduced survival compared to patients with low levels of the two genes, and lung SCC patients with high levels of *TNFRSF1A* and *CDK6* had significantly reduced survival compared to patients with low levels of *TNFRSF1A* (Fig. 2h, middle and right). Together, these results demonstrate the importance of increased TNFR1 and its targets for lung SCC development in humans and mice.

### Silencing TNFR1 reverses dedifferentiated spindle cell carcinoma to well-differentiated lung SCC

**Fig. 3:**
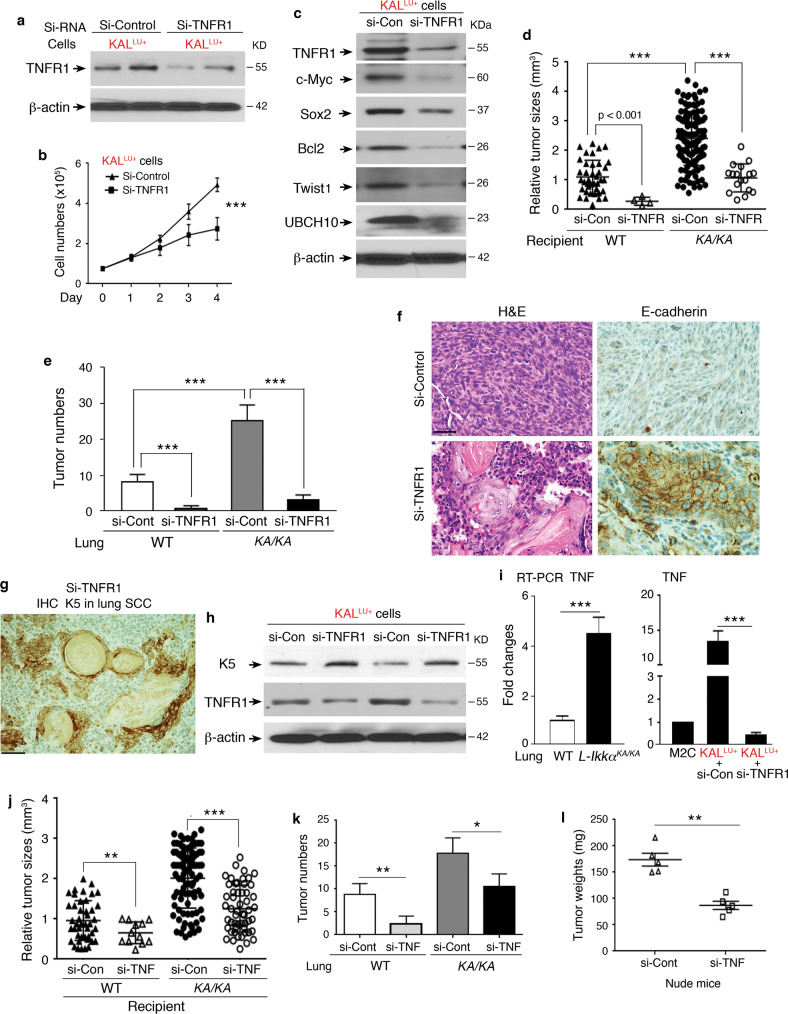
Silencing TNFR1 expression reverses dedifferentiated spindle cell carcinoma to well-differentiated SCC. **a** Immunoblotting analysis showing TNFR1 levels in KAL^LU+^ cells treated with TNFR1 siRNA (si-TNFR1) or si-Control. β-actin, a protein-loading control. **b** Growth curve of KAL^LU+^ cells treated with si-TNFR1 or si-Control. Mean ± SD (three repeats); ***P < 0.001; Two-way AVOVA statistical test. **c** Immunoblotting analysis shows indicated protein levels in KAL^LU+^ cells treated with si-Control (si-Con) or si-TNFR1. β-actin, a protein-loading control. **d**, **e** Comparing tumor sizes (**d**) and numbers (**e**) derived from KAL^LU+^ cells treated with si-Control (si-Con) or si-TNFR1 in WT and *KA/KA* mice (recipient, *n* = 5/group). Each symbol represents a tumor. Mean ± SD; ***P < 0.001; Student’s *t*-test. **f** H&E-stained and E-cadherin IHC-stained sections of tumors derived from KAL^LU+^ cells treated with si-Control or si-TNFR1 in *KA/KA* mice (recipient). Scale bar: 40 μM. **g** K5 IHC staining for lung SCCs derived from si-TNFR1 KAL^LU+^ cells in *KA/KA* mice at 1.5 months after cell injections. Scale bar: 50 μM. **h** Immunoblotting analysis showing the levels of K5 and TNFR1 in KAL^LU+^ cells treated with si-Control and si-TNFR1. β-actin, a protein-loading control. **i** RT-PCR analysis showing TNF expression in WT and *L-KA/KA* lungs (left) and in M2C and KAL^LU+^ cells (right) treated with si-Control (si-Con) and si-TNFR1 (*n* = 3/group). Mean ± SD (three repeats per group); ***P < 0.001; Student’s *t*-test. **j**, **k** Comparing tumor sizes (**j**) and tumor numbers (**k**) derived from KAL^LU+^ cells treated with si-Control (si-Con) or si-TNF in WT and *KA/KA* mice (*n* = 5/group). Mean ± SD; *P < 0.05; **P < 0.01; ***P < 0.001; Student’s *t*-test. **l** Weights of tumors isolated from nude mice receiving subcutaneous injections of KAL^LU+^ cells treated with si-Control (si-Cont) and si-TNF RNA (*n* = 5/group). Mean ± SD; **P < 0.01; Student’s *t*-test.

To demonstrate that TNFR1 expression in cell lines determines SCC differentiation, we used TNFR1 siRNA to silence TNFR1 in KAL^LU+^ cells (Fig. 3a). TNFR1 downregulation inhibited cell growth and reduced c-Myc, Sox2, Bcl2, Twist1, and UBCH10 protein levels (Fig. 3b, c). Tail-vein injected si-TNFR1 KAL^LU+^ cells generated significantly fewer and smaller SCCs in the lungs of WT and *KA/KA* mice than did KAL^LU+^ cells (Fig. 3d, e). si-RNA-induced gene silencing can last 5-10 days in cells [30]. To ensure that this approach was well performed, we verified si-RNA-induced TNFR1 reduction before cell injection. Notably, there were more lung tumors in *KA/KA* mice compared to WT mice, likely *KA/KA* lungs are highly inflamed [3], which promotes the tumorigenesis. The effect of TNFR1 on the tumor microenvironment is not covered here but will be discussed in future studies.

We failed to detect E-cadherin, a cellular marker that is expressed on the surface of well-differentiated SCCs [31], in the spindle cell carcinomas generated by KAL^LU+^ cells (Fig. 3f); however, E-cadherin expression was detected on the surface of the well-differentiated SCC cells generated by si-TNFR1 KAL^LU+^ cells (Fig. 3f). Likewise, spindle cell carcinomas generated by KAL^LU+^ cells lacked K5 expression (Fig. 2g), while si-TNFR1 KAL^LU+^ cell–generated SCCs re-expressed K5, as examined by IHC staining (Fig. 3g). Consistently, K5 protein levels were higher in si-TNFR1 KAL^LU+^ cells than in KAL^LU+^ cells (Fig. 3h). This indicates that TNFR1, associated with stemness, determines the differentiation program of SCC cells. Overexpression of TNFR1 drives a dedifferentiated phenotype.

TNF is a ligand for TNFR1 [32], so we examined its expression in mouse models and cell lines. TNF was more highly expressed in *KA/KA* lungs than in WT lungs and in KAL^LU+^ cells than in M2C cells (Fig. 3i, left). Si-RNA mediated downregulation of TNFR1 in KAL^LU+^ cells reduced TNF expression (Fig. 3i, right). To determine whether TNF is involved in TNFR1-regulated tumor development, we used TNF siRNA to silence TNF in KAL^LU+^ cells. These cells generated significantly fewer and smaller (based on size or weight) tumors than KAL^LU+^ cells in *KA/KA*, WT, and nude mice (Fig. 3j–l). Moreover, the expression of *TNFRSF1A* and *TNF* was positively correlated in human lung SCCs (Fig. S3). Thus, TNFR1 levels regulate TNF expression and TNF is a modulator in the TNFR1-mediated tumorigenic loop.

### Delineation of the tumorigenic TNFR1 pathway

**Fig. 4:**
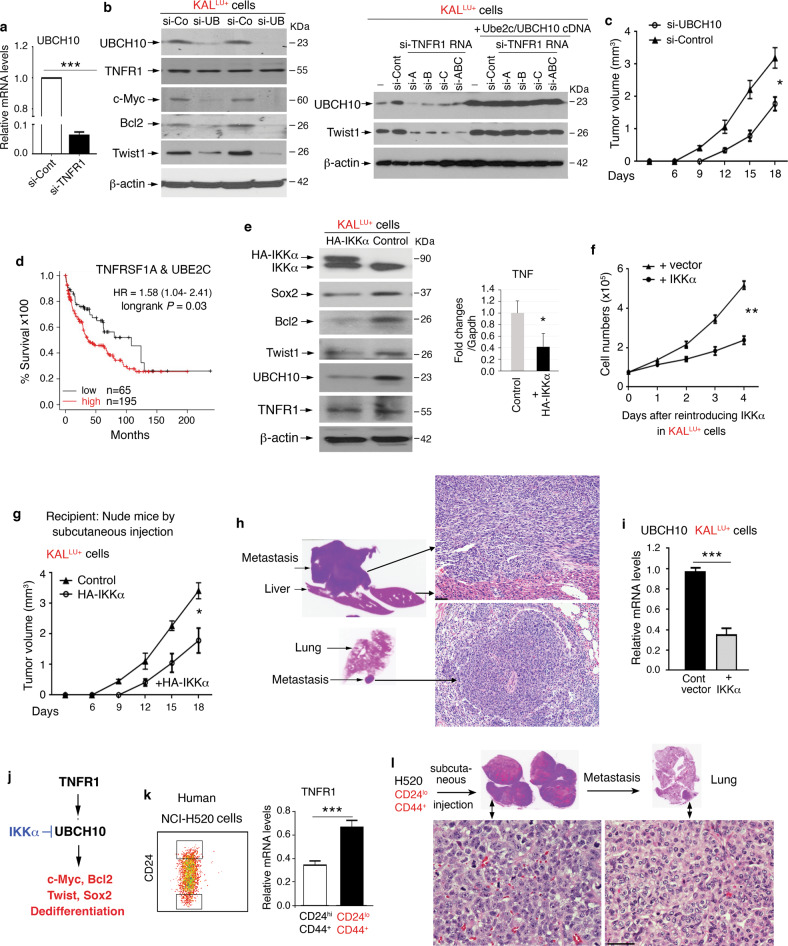
Overexpressed TNFR1-mediated signaling for tumorigenesis. **a** RT-PCR showing UBCH10 expression in KAL^LU+^ cells treated with si-Control (si-Cont) and si-TNFR1. ***P < 0.001; Student’s *t*-test (mean ± SD of three samples per group). **b** Left: Immunoblotting analysis showing levels of the indicated proteins in KAL^LU+^ cells treated with si-Control (si-Con) and si-UBCH10 (si-UB). β-actin, a protein-loading control. Right: Immunoblotting analysis showing Twist and UBCH10 levels in KAL^LU+^ (-), si-TNFR1 RNA-treated KAL^LU+^ cells (si-TNFR1 RNA including si-A, si-B, si-C, and si-ABC), and si-TNFR1 RNA-treated KAL^LU+^ cells with reintroduced Ube2c/UBCH10 cDNA. β-actin, a protein-loading control. **c** Left: Comparison the growth of tumors, derived from KAL^LU+^ cells treated with si-Control or si-UBCH10, in nude mice (*n* = 5/group). Mean ± SD; *P < 0.01; two-way AVOVA test. **d** Survival curves of lung SCC patients with high TNFRSF1A and UBE2C levels or low TNFRSF1A levels. Lung SCC patient cohorts were obtained from Kaplan–Meier plotter. **e** Left: Immunoblotting analysis showing levels of K5, E-cadherin, and UBCH10 in KAL^LU+^ cells overexpressing a control or HA-IKKα vector. β-actin, a protein-loading control. Right: RT-PCR examines TNF expression in KAL^LU+^ cells and KAL^LU+^ cells overexpressing HA-IKKα with. Mean ± SEM (three repeats per group). **P < 0.01; Student’s *t*-test. **f** Growth curves of KAL^LU+^ cells that were transfected with a control vector or HA-IKKα vector (*n* = 5/group). Mean ± SD; **P < 0.01; two-way AVOVA statistical test. **g** The sizes of tumors obtained from KAL^LU+^ cells treated with the control or HA-IKKα vector (2 × 10^5^ cells per mouse) were subcutaneously injected into nude mice (*n* = 5/group). Tumor growth was analyzed by a grouped two-way AVOVA statistical test; Mean ± SD; *P < 0.05. **h** Left: Images showing a liver (top) and lung (bottom) with metastases (Fig. S4a shows H&E histological images). Right: H&E images of liver and lung metastases induced by arrows. Scale bar: 40 μM. **i** RT-PCR showing UBCH10 mRNA levels in KAL^LU+^ cells transfected with IKKα or control vectors (*n* = 3/group). Mean ± SD; ***P < 0.001; Student’s *t*-test. **j** A working model for the TNFR1-UBCH10 pathway predicted to increase c-Myc, Bcl2, Twist1, and Sox2 expression and SCC dedifferentiation. IKKα inhibits UBCH10 expression. Arrow, enhancement; two lines, inhibition. **k** Human H520 SCC cell line was selected with stem cell markers and used to generate two sub-populations (left). H520 (CD24^lo^CD44^+^) cells express elevated TNFR1 levels compared to H520 (CD24^hi^CD44^+^) cells. Mean ± SD (three repeats per group); ***P < 0.001; Student’s *t*-test. **l** H520 (CD24^lo^CD44^+^) cells generated tumors induced by subcutaneous injections and metastasis to lungs in nude mice (*n* = 5). Scale bar: 40 μM.

Silencing TNFR1 also reduced UBCH10 mRNA levels (Fig. 4a) and silencing UBCH10 reduced c-Myc, Bcl2, and Twist1 without significantly altering TNFR1 levels (Fig. 4b, left). To determine whether UBCH10 is a target of TNFR1, we used si-TNFR1 RNA to downregulate Twist1 and UBCH10 levels in KAL^LU+^ cells and showed that reintroduced UBCH10 restored Twist1 expression in si-TNFR1-treated KAL^LU+^ cells, indicating that UBCH10, which controls Twist1 expression, is a downstream effector of TNFR1 (Fig. 4b, right, and S4a, left). Moreover, si-RNA-mediated reduction of UBCH10 in KAL^LU+^ cells attenuated their ability to form tumors in nude mice (Fig. 4c). This may also be true in lung SCC patients as high levels of *TNFRSF1A* and *UBE2C* correlate with poor survival (Fig. 4d).

KAL^LU^ cells express reduced IKKα [3]. To determine the role of IKKα in the tumorigenesis, we restored HA-IKKα expression in KAL^LU+^ cells and observed decreased Sox2, Bcl2, Twist1, and UBCH10 levels in the absence of altered TNFR1 levels, as examined by immunoblotting as well as reduced TNF expression determined by RT-PCR (Fig. 4e, left and right, and Fig. S4a, right). Thus, IKKα downregulates UBCH10 expression. Increasing expression of IKKα in KAL^LU+^ cells inhibited their proliferation (Fig. 4f), dampened tumorigenesis, and blocked metastases to the liver and lungs in nude mice (Fig. 4h and Fig. S4b). IKKα reintroduction also reduced UBCH10 mRNA levels in KAL^LU+^ cells (Fig. 4i). Thus, IKKα inhibits UBCH10, c-Myc, Bcl2, Twist, and Sox2 expression (Fig. 4j), which is opposite to TNFR1.

To evaluate the role of TNFR1 in human SCC cells, we isolated human H520 SCC cells [33] with stem cell markers (Fig. 4k, left). H520 cells with CD24 ^lo^CD44^+^ stemness markers expressed increased TNFR1 (Fig. 4k, right, and S4c), and generated tumors and metastases to the liver and lungs in nude mice after being subcutaneously injected (Fig. 4l and Fig. S4d), while H520 cells expressing CD24^hi^CD44^+^ markers failed to generate tumors. Thus, TNFR1 levels in human SCC cells correlate with their ability to form tumors.

### UBCH10 is required for SCC cell dedifferentiation; NF-κB is involved in regulation of UBCH10 expression

**Fig. 5:**
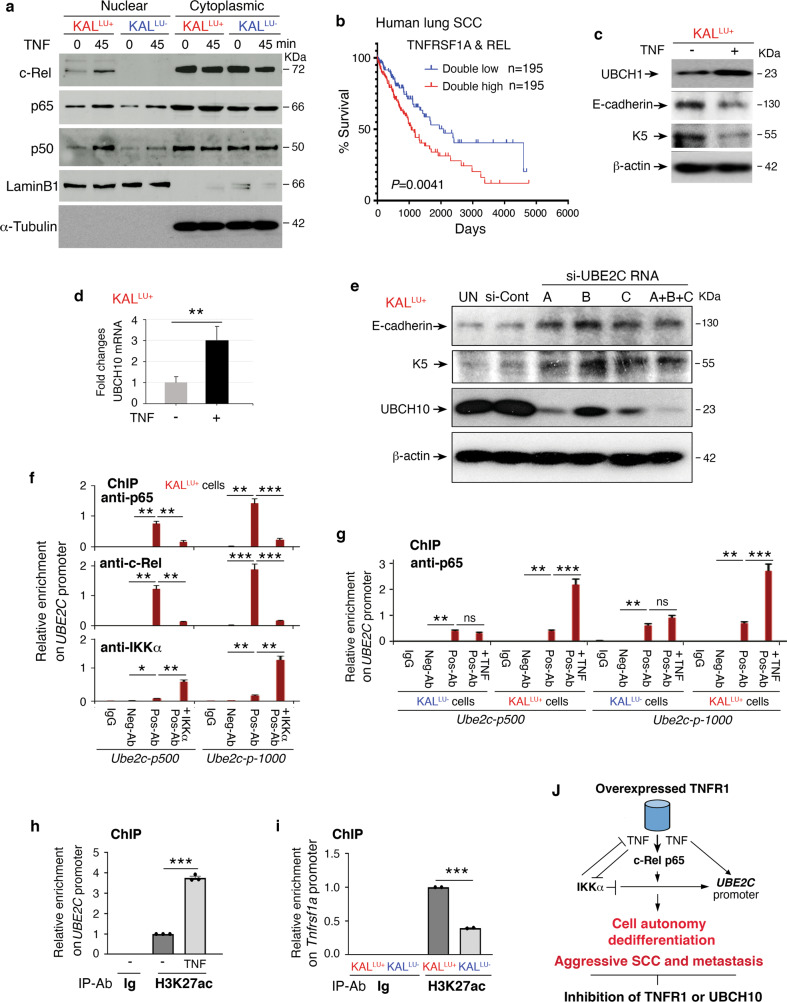
The mechanism for regulation of UBE2C expression. **a** Immunoblotting showing levels of the indicated nuclear and cytoplasmic protein levels in KAL^LU+^ and KAL^LU-^ cells following TNF (10 ng/mL) stimulation. α-Tubulin, a cytosol protein-loading control. **b** Survival curves of human lung SCC expressing double-high TNFRSF1A and REL levels compared to low TNFRSF1A and REL levels. These data were obtained from the Kaplan–Meier plotter. **c** Immunoblotting showing UBCH10, E-cadherin, and keratin 5 (K5) levels in KAL^LU+^ cells treated with and without TNF (10 ng/mL) treatment for 60 min. β-actin, a protein-loading control. **d** RT-PCR shows UBCH10 expression in KAL^LU+^ cells treated with TNF (10 ng/mL). Mean ± SEM (three repeats per group). **P < 0.01; Student’s *t*-test. **e** Immunoblotting showing levels of the indicated protein levels in KAL^LU+^ cells following treatment with three different si-UBE2C RNAs (A, B, C, and A + B + C). β-actin, a protein-loading control. **f** ChIP assay showing the enrichment of p65, c-Rel, and IKKα associated with the *Ube2c* promoter (p) on regions upstream of −500 bp and −1000 bp in KAL^LU+^ cells, immunoprecipitated with p65, c-Rel, and IKKα antibodies. The enrichment was compared to input levels. anti- or Ab, antibody; Neg-Ab, negative-control antibody (Ig); Pos-Ab, positive experimental anti-p65, c-Rel, and IKKα antibody; +IKKα, overexpressed HA-IKKα in KAL^LU+^ cells. **g** ChIP assay showing the enrichment of p65 associated with the *Ube2c* promoter (p) on regions upstream of −500 bp and −1000 bp, immunoprecipitated with p65 antibody in KAL^LU-^ and KAL^LU+^ cells. The enrichment was compared to input levels. anti- or Ab, antibody; Neg-Ab, negative-control antibody; Pos-Ab, positive p65 antibody; TNF, TNF (10 ng/mL) treatment for 45 min. **h** ChIP assay showing the enrichment of H3K27ac on the *Ube2c* promoter (−500 bp) in KAL^LU+^ cells treated with and without TNF (10 ng/mL). **i** ChIP assay showing the enrichment of H3K27ac on the *Tnfrsf1a* promoter (−500 bp) in KAL^LU-^ and KAL^LU+^ cells. **j** A working model for how overexpressed TNFR1 promotes and IKKα inhibits *Ube2c* gene expression, resulting in SCC cell dedifferentiation, aggressive tumors, and metastasis. Silencing TNFR1 or UBCH10 inhibits TNFR1-enhanced tumorigenesis. Arrow, promotion; crossed lines, inhibition.

Because TNFR1 regulates oncogenic UBCH10 expression, we tested whether its ligand TNF, which is also regulated by TNFR1, elevates UBCH10 expression to alter the differentiation of SCC cells. We found that TNF treatment of KAL^LU+^ cells for 45 min increased the levels of nuclear c-Rel, p65, and p50, three subunits of NF-κB transcription factor family (Fig. 5a). In contrast, TNF treatment of KAL^LU-^ cells induced a slight increase in nuclear p65 and p50 levels but did not affect nuclear c-Rel levels in (Fig. 5a). In addition, the regulatory element of the *UBE2C* promoter contains c-Rel binding consensus sites [19, 34]. Patients with lung SCC expressing both high *TNFRSF1A* and *REL* levels showed reduced survival compared to lung SCC patients expressing both low levels of these genes (Fig. 5b). Also, patients with lung SCC expressing high *TNFRSF1A* and *RELA* levels showed reduced survival compared to lung SCC patients expressing low *TNFRSF1A* levels (Fig. S5a). These suggest that increased expression of *TNFRSF1A*, *REL* and *RELA* is relevant to human lung SCC.

Furthermore, TNF treatment enhanced UBCH10 protein and mRNA levels and decreased E-cadherin and K5 expression, which correlates with dedifferentiation, in KAL^LU+^ cells (Fig. 5c, d), but the treatment did not significantly alter K5 and E-cadherin levels in KAL^LU-^ cells (Fig. S5b). On the other hand, silencing UBE2C, which encodes UBCH10, upregulated well-differentiated SCC marker E-cadherin and keratin 5 (K5) protein levels in KAL^LU+^ cells (Fig. 5e). These results suggest that UBCH10 is a regulator for SCC differentiation. Like TNFR1, TNF can induce expression of UBCH10 and dedifferentiation markers in SCC cells possibly via NF-κB transcriptional regulation in high-TNFR1 KAL^LU+^ cells because these cells are sensitized to TNF.

To test whether TNF/TNFR1 regulates UBCH10 expression through NF-κB transcriptional activation, we performed chromatin immunoprecipitation (ChIP) experiments. Three c-Rel consensus sites were identified at positions −450 bp, −920 bp, and −1,110 bp in the *Ube2c* promoter. ChIP identified enrichment of c-Rel and p65 transcription factors on the *Ube2c* promoter at ~−500 bp and −1000 bp in KAL^LU+^ cells (Fig. 5f). Interestingly, reintroduced IKKα in KAL^LU+^ cells correlated with reduced enrichment of c-Rel and p65 on the *Ube2c* promoter (Fig. 5f), indicating that NF-κB and IKKα play opposing roles in regulating *Ube2c* expression. However, we did not observe enrichment of c-Rel and IKKα at the distal promoter (−1500 bp and −5000 bp) of *Ube2c* (Fig. S5c), which was used as a negative control.

Consistent with the hypothesis that TNFR1 stimulates expression of *Ube2c* via NF-κB, we found that TNF treatment significantly enhanced the enrichment of p65 on the *Ube2c* promoter at ~−500 bp and −1000 bp in KAL^LU+^ cells but not in KAL^LU-^ cells (Fig. 5g). We did not detect p65 enrichment on the distal promoter (−1500 bp and −5000 bp) of *Ube2c* after TNF stimulation (Fig. S5d). TNF treatment also significantly enhanced the enrichment of p65 on the *UBE2C* promoter at ~−500 bp and −1000 bp but not at the distal *UBE2C* promoter (−1500 bp and −5000 bp) in two human lung SCC cell lines: NIH520 and SW900 (Fig. S5e). To confirm that TNF promotes transcriptional activation of the *Ube2c* locus in KAL^LU+^ cells, we examined the enrichment of H3K27ac [35], which is a marker for acetylation of the lysine (K) residue at position 27 and is associated with transcriptionally active chromatin, on the *Ube2c* promoter. KAL^LU+^ cells were treated with TNF and subjected to ChIP using an anti-H3K27ac antibody and PCR primers flanking the −500 bp *Ube2c* promoter region. The levels of H3K27ac enrichments were much higher in TNF-treated than in control cells (Fig. 5h). These results suggest that TNF/TNFR1 promotes UBCH10 expression by activating the *Ube2c* promoter. In addition, to understand why TNFR1 levels are higher in KAL^LU+^ cells than in KAL^LU-^ cells, we conducted ChIP with an anti-H3K27ac antibody and PCR primers flanking the *Tnfrsf1a* promoter at the -500 bp position. H3K27ac enrichments was more abundant in KAL^LU+^ cells than in KAL^LU-^ cells (Fig. 5i), indicating that the transcriptional activity of the *Tnfrsf1a* locus is higher in KAL^LU+^ cells than in KAL^LU-^ cells.

Collectively, these findings suggest that elevated TNFR1 promotes expression of UBCH10 and its targets in KAL^LU+^ cells by TNF-induced NF-κB activation, leading to dedifferentiated and aggressive SCC and metastases (Fig. 5j). In contrast, IKKα antagonizes TNFR1-enhanced tumor progression and metastasis. We propose that inhibiting TNFR1 or UBCH10 is a potential therapeutic strategy for lung SCC.

## Discussion

We identified increased TNFR1 levels in a large number of human lung SCCs, in particular, it was highly expressed in the SCC epithelial cells. Thus, elevated TNFR1 expression may influence human lung SCC development in a cell-autonomous manner. We investigated this using animal models and found that the TNFR1 level determines SCC cell differentiation via a TNFR1-UBCH10 axis. UBCH10, a cancer-related E2 ubiquitin-conjugating enzyme, is highly expressed in human lung SCCs and upregulates the expression of Twist1, c-Myc, and Sox2 [27, 28]. Consistently, we found that high expression levels of the *TNFRSF1A* and *UBE2C* or *TWIST1* genes in lung SCCs correlate with poor patient survival.

Human and mouse lung SCC cells express stem cell markers [3, 36]. Here, we revealed that TNFR1 expression correlates with, and in fact controls, stemness levels. Importantly, the levels of K5 and E-cadherin (a cell-cell junction regulator and a marker for well-differentiated SCC [31]) were higher in mouse TNFR-low SCC cells than in TNFR1-high cells. TNFR-low cells formed well-differentiated SCCs, while TNFR1-high cells formed dedifferentiated spindle cell carcinoma and metastasis in vivo. The spindle cell carcinoma, the final stage of SCC, loses expression of the K5 basal cell marker and of E-cadherin but exhibits EMT characters [37, 38]. Thus, these findings establish a correlation between the levels of TNFR1 in K5 positive epithelial cells and the differentiation status of mouse SCC cells. This was also true for human cell lines, as we demonstrated that the oncogenic activity of two human SCC lines correlated with their TNFR1 levels.

We established that TNFR1 levels can control SCC differentiation status using transgenic mice. Tg-K5.TNFR1;*KA/KA* mice developed poorly differentiated lung SCC and metastasis compared to well-differentiated lung SCCs detected in *KA/KA* mice. In contrast, *KA/KA;Tnfrst1a*^*-/-*^ mice failed to generate spontaneous lung SCCs. Of note, we previously identified multiple elevated oncogenes and decreased tumor suppressors in *KA/KA* lung SCCs, but we did not observe an increase in AKT and PIK3, which are important for human lung cancer development and therapy [6]. Here, our discovery that TNFR1 expression appears to affect tumor outcomes in patients and in mice highlights a new pathway for lung SCC pathogenesis.

Because *KA/KA* mice develop an autoimmune disease with systemic autoinflammation [3], which can alter tumorigenesis in a non-cell-autonomous manner, we used two SCC cell lines (KAL^LU-^ and KAL^LU+^) to study how epithelial cell-autonomous expression of TNFR1 affects lung SCC pathogenesis. KAL^LU+^ cells were more stem cell-like and expressed higher levels of TNFR1, UBCH10, Sox2, Twist1, and Bcl2 than KAL^LU-^ cells. KAL^LU+^ cells generated dedifferentiated spindle cell carcinomas in vivo, but silencing TNFR1 in these cells reduced the expression of these molecules and inhibited aggressive tumorigenesis and metastasis. These findings obtained from the different mouse model systems and SCC patients are consistent. Interestingly, we observed multiple nonspecific bands in cells and SCCs that highly expressed TNFR1 levels detected using immunoblotting with an anti-TNFR1 antibody (data not shown). According to existing reports [23, 39, 40], TNFR1 can form oligomers with different partners or be cleaved by metalloproteinases. Whether these different TNFR1 oligomers or short TNFR1 forms impact lung carcinogenesis remains to be examined in the future.

The link between TNFR1 and NF-κB signaling in SCCs is a novel observation. Previously, we did not observe higher nuclear p65 levels in *KA/KA* lung epithelial cells than in WT lung epithelial cells [3]. Nor did we observe elevated nuclear p65 in TNF-stimulated KAL^LU^ cells [3], which are the parental cells of the newly established KAL^LU-^ and KAL^LU+^ lines. Here, we showed that TNF treatment induced NF-κB nuclear translocation in KAL^LU+^ cells but not in KAL^LU-^ cells. The *Ube2c* promoter contains c-Rel consensus binding sites [19, 34] and TNF treatment stimulated c-Rel, p65, and H3K27ac enrichment on the *Ube2c* promoter in KAL^LU+^ cells but not in KAL^LU-^ cells. TNF elevated UBCH10 levels and reduced K5 and E-cadherin expression in KAL^LU+^ cells, and si-RNA mediated silencing of TNF in these cells attenuated tumorigenesis. Thus, KAL^LU+^ cells that have elevated TNFR1 are highly responsive to TNF stimulation, which can further upregulate *Ube2c* transcription. In addition, the transcriptional activation maker, H3K27ac, on the *Tnfrsgf1a* locus was higher in KAL^LU+^ cells than in KAL^LU-^ cells. Thus, an epigenetic mechanism regulates TNFR1 expression.

Previously, we showed that spontaneous *KA/KA* lung SCC development is highly associated with inflammation and macrophage infiltration, but reintroducing IKKα into K5-positive lung epithelial cells prevents lung SCC development in *KA/KA* mice [3], suggesting that the K5-positive cells are the target of tumorigenesis. In this study, we found that IKKα, in contrast to TNFR1/TNF, negatively regulated UBCH10 expression without affecting TNFR1 levels. Consistent with this, the enrichment levels of IKKα and c-Rel/p65 on the *Ube2c* promoter were reciprocal. Reintroducing IKKα into KAL^LU+^ cells inhibited their ability to form tumors and metastases. Thus, IKKα is a negative regulator that can oppose the pro-tumor potential of TNFR1 in a cell-autonomous fashion.

Of note, reintroduced IKKα repressed TNF and UBCH10 expression in KAL^LU+^ cells, while TNF treatment enhanced UBCH10 expression but reduced SCC differentiation markers. Previously, we reported that IKKα downregulates TNF amounts in lung ADC cells at the transcriptional level [41] and that TNF suppresses IKKα expression in human SCC cells [21]. Thus, a low level of IKKα maintains continuous TNF expression in an autoregulatory loop that impacts tumorigenesis. It is likely that IKKα regulates TNF expression via a common mechanism in lung SCC and ADC. Furthermore, IKKα is a central regulator for keratinocyte terminal differentiation and proliferation [42–44]. Reduced IKKα accelerates well-differentiated papilloma development and loss of two *Ikka* alleles promotes the transition from well-differentiated skin papilloma to poorly differentiated SCC associated with increased TNF expression [10]. Elevated IKKα levels in the skin inhibit the incidence of skin tumors and metastasis [11, 20]. These findings indicate that IKKα targets the TNF/TNFR1-mediated pathway in preventing SCC development, progression, and metastasis.

Furthermore, *Ikka*-deficient lung ADCs contribute to a pro-tumor immunosuppressive milieu by upregulating TNF expression, which activates TNFR2/NF-κB/Foxp3 signaling to enhance the conversion of CD4 T cells to Foxp3 Treg cells in *Kras*^*D12G*^*Ikka*^*ΔLU*^ mice that have a wild-type immunological background before Ras activation and *Ikka* ablation in the lungs [41, 45]. Unlike *Kras*^*D12G*^*Ikka*^*ΔLU*^ mice, *KA/KA* mice develop autoreactive T cell-mediated autoimmune syndromes due to defective medullary thymic epithelial cell development caused by this *KA/KA* germline mutation [21, 22]. Reduced Foxp3 Treg numbers were detected in the thymus, spleens, and lungs of *KA/KA* mice and Foxp3 cell injection did not improve *KA/KA* lung conditions [21] (data not shown). Thus, elevated TNF amounts generated by *KA/KA* lung SCCs are not a major driver that can determine the tumor-microenvironment’s influence. On the other hand, increased macrophage infiltration is required for lung SCC development in *KA/KA* mice [3]. Currently, we are investigating the relationship between macrophages and autoreactive CD4 T cells in lung SCC pathogenesis. Together, these data highlight that TNF can facilitate tumorigenesis via the TNFR1 or TNFR2 pathway, depending on the cell types and microenvironments involved.

In summary, TNFR1 stimulates UBCH10 expression via NF-κB signaling leading to expression of UBCH10 target genes in lung tumor-epithelial cells. The TNFR1-UBCH10 axis establishes a cascade that drives well-differentiated SCCs into dedifferentiated spindle cell carcinomas, the most aggressive SCC and one with EMT features. Silencing TNFR1 in SCC cells inhibits cell proliferation, reduces UBCH10, Twist1, Sox2, and c-Myc levels, converts spindle cell carcinomas into well-differentiated SCCs, dampened tumor burden, and blocked metastasis. Thus, TNFR1 inhibitors could potentially be used for differentiation therapy to treat lung SCC.

## Materials and methods

### Animal experiments and human samples

All mice used in this study were cared for in accordance with the guidelines of the Institutional Animal Care and Use Committee (IACUC), National Institutes of Health, and all animal experiments were approved by an IACUC (protocols 11–051, 11–052, 14–051, 14–052, 20–051, and 20–052). *L-Ikkα*^*KA/KA*^ (*KA/KA*) mice [3] and WT mice on an FVB background were used in this study. FVB *KA/KA;Tnfrsf1a*^*-/-*^ mice were generated by crossing *KA/KA* with *Tnfrsf1a*^*-/-*^ mice (B6.129-*Tnfrsf1a*^tm1Mak^/J, from The Jackson Laboratory). Female nude mice (Strain #:000819) for the experiments were purchased from the Jackson Laboratory. PCR primers for genotyping the *Tnfrsf1a*^*-/-*^ mice were: 5'-TGT GAA AAG GGC ACC TTT ACG GC-3' (wt), 5'-GGC TGC AGT CCA GGC ACT GG-3' (common), and 5'-ATT CGC CAA TGA CAA GAC GCT GG-3' (mutant). The Frederick National Laboratory for Cancer Research generated the Tg-K5.TNFR1 transgenic mice. All experimental mice had an FVB background and the experimental animals used for this study were randomly picked up in a blinding manner. Human lung tumors were obtained from Dr. David Schrump in the Thoracic Surgery Branch, National Cancer Institute. All human samples used in this study were procured on National Institutes of Health Internal Review Board approved protocol 06-C-0014 (NCT 00242723), and informed consent was obtained from all patients. Human normal lung tissue lysates were obtained from Abcam (ab43320, ab42178, and ab42527, Cambridge, MA). The tissue array (LC991 088) containing 32 human SCCs with their proximal and distal adjacent lung tissues was purchased from US Biomax, Inc. (Rockville, MD). Human lung squamous cell carcinoma cell lines NCI-H520 (H520) and SW900 (HTB-59) were purchased from American Type Culture Collection (ATCC, Manassas, VA).

### Transgenic mice

A mouse TNFR1 full-length cDNA fragment (1,365 bp) in the CMV-Myc Flag TNFR1 vector (MR226545, OriGene, Rockville, MD) was purified after being digested with *KpnI* and *PmeI*. This TNFR1 cDNA fragment was subcloned into the BK5 vector [46] that was digested using the enzyme *XhoI*. Sequencing was used to confirm the insertion of the TNFR1 cDNA fragment (1,365 bp) into the BK5 vector. The construct was linearized with *KpnI* digestion. The Transgenic Mouse Model Laboratory, Leidos Biomedical Research, Inc., Frederick National Laboratory for Cancer Research (Frederick, MD), generated the transgenic mice from an FVB background and confirmed the transgenic mice by Southern blotting with a TNFR1 cDNA probe. PCR primers for genotyping the transgenic mice were: 5´-TCA GGG GTG TTG TTT AGA ATG G-3´ and 5´-CAA TAA GAA TAT TTC CAC GCC A-3´ were used to genotype the transgenic mice.

### Antibodies

Antibodies used in this study included IKKα (IMG-136A) from IMAGENEX; Lamin B (sc-6216), cytokeratin 5 (K5, sc-17090), p50 (sc-1109), c-myc (sc-764), and IgG (sc-2025) from Santa Cruz Biotechnology; p65 (8242), c-Rel (12707), and E-cadherin (24E10) from Cell Signaling Technology; Sox2 (245610) from R&D Systems; Twist1 (T-6451) and β-actin (A-5441) from Sigma-Aldrich; TNFR1 (ab111119 and ab19139) and histone H3 (acetyl K27, ab4729) from Abcam; UBCH10 (NBP2-20782) from Novus Biologicals; K5 (PRB-160P) from Babco; α-Tublin (ab4074) and histone H3 (acetyl K27, ab4729) from Abcam.

### Histopathology, immunoblot analysis, and immunohistochemical and immunofluorescent staining

The Histology and Tissue Core Facility at the Frederick National Laboratory for Cancer Research routinely prepared paraffin sections of mouse organs and performed hematoxylin and eosin staining and immunohistochemical staining for TNFR1, K5, E-cadherin, loricrin, and involucrin. Cell lysates (15 µg) or protein extracts from the tissues (20 µg) were separated on acrylamide gels and proteins detected using immunoblotting with specific antibodies, as previously described [47]. Human array sections containing 49 lung SCCs, 50 cancer-adjacent tissues, and 50 normal lung tissues (BC04118, US Biomax) were immunohistochemically stained with an anti-TNFR1 antibody (ab111119, Abcam) by Histoserv, Inc. (Germantown, MD). The Histology and Tissue Core Facility at the Frederick National Laboratory for Cancer Research analyzed the array results. Immunofluorescent staining (IF) for UBCH10 in the paraffin sections of mouse lungs and lung tumors was performed as previously described [3].

### Preparation of nuclear protein from mouse lung SCC KAL^LU^ cells

Cultured cells were washed twice with cold PBS, then were collected by spinning at 3000 rpm for 5 min. Next, 200–500 μL of cytoplasmic extract buffer (10 mM KCl, 10 mM Hepes, [pH 7.9], 3 mM MgCl_2_, 1.0% NP-40) were added to the cell pellets. Pellets were gently mixed, kept on ice for 5 min, and then centrifuged at 6000 rpm for 5 min. The supernatants were collected as the cytoplasmic extract. Pellets were washed once with 500 μL of cytoplasmic extract buffer, suspended in 100–200 μL of nuclear extract buffer (400 mM KCl, 10 mM Hepes [pH 7.9], 3 mM MgCl2, 1.0% NP-40), gently mixed on ice for 5 min, and centrifuged at 14,000 rpm for 5 min. Supernatants were collected as the nuclear extract.

### Cell isolation and flow cytometry

Isolation of murine lung SCC stem cells was performed by flow sorting on a BD FACSAria using Sca1 (Ly6A/E, clone D7) and CD24 (clone M1/69) fluorescence-conjugated antibodies from BD Pharmingen. For flow sorting the human SCC stem cells from the line, the antibodies against CD24 (clone ML5, BioLegend) and CD44 (clone IM7, eBioscience) were used. Data was analyzed using FlowJo 3.2 version software by Tree Star. In addition, we used a standard procedure of flow cytometry to analyze TNFR1 expression in cell lines. In brief, KAL^LU-^ and KAL^LU+^ cells were harvested and resuspended in 50 μl PBS containing 0.5% BSA buffer in the presence of a mouse TNFR1 antibody (113005, Biolegend) for 30 min at 4 °C in a dark place. Data acquisition was performed using a FACSCanto II device (BD Biosciences) using FlowJo software (Tree Star).

### Isolation of genomic DNA and PCR

After *KA/KA* and WT mice received injections of H520 cells and KAL^LU^ cells, genomic DNA was isolated from lung tumors by using genomic DNA extraction buffer (50 mM Tris, 1 mM EDTA, 0.5% SDS, 1 mg/mL Proteinase K), followed by phenol–chloroform extraction. DNA concentrations and purity were measured using a NanoVue (GE Healthcare Life Sciences), and DNA integrity was confirmed by gel electrophoresis. PCR was performed to confirm that the lung tumors were derived from the injected H520 cells using primers (with the primers 5'-ATTTAATCTCTCCAGCAAGTTG-3' and 5'-TAATTCATTAATAATTACTTACACAG-3′) for the intron sequences of the human IKKα gene. PCR conditions used were: one cycle at 95 °C for 4 min; 33 cycles at 95 °C for 30 s, 57 °C for 30 s, and 72 °C for 45 s; and one cycle at 72 °C for 10 min.

### Microarray

Total RNA was isolated from cultured cells (KAL^LU+^ cells and KAL^LU-^ cells) using the TRIzol Reagent (Invitrogen). RNA of sufficient quality (by Bioabalyzer) was used for the Affymetrix GeneChip Mouse Genome 430 2.0 Array at the Laboratory of Molecular Technology (Cancer Research Technology Program, Frederick National Laboratory for Cancer Research). RNA samples (100 ng of each) were labeled with the Affymetrix IVT Express labeling kit (Affymetrix, Santa Clara, CA) and hybridized to the Mouse Genome 430 2.0 array according to the manufacturer’s suggested protocol. GeneChips were scanned on the Affymetrix GeneChip Scanner 3000, and data were collected using Affymetrix AGCC software. Microarray data were analyzed by faculty from the National Cancer Institute Center for Cancer Research Bioinformatics Core (Bethesda, MD), using Partek Genomics Suite software. Accession numbers for the original microarray data (accession no. GSE65291) were deposited at the NCBI Gene Expression Omnibus.

### RT-PCR

Total RNA was isolated from lung tissues or cultured cells using the TRI Reagent (Molecular Research Center, Inc., Cincinnati, OH). cDNA was synthesized with a SuperScript^®^III First-Strand kit (18080-051, Invitrogen, CA). PCR primers were used to detect TNFR1 cDNA (5'-AGTGCACGAAGTTGTGCCTAC-3' and 5'- TGATAGGGTGGTGCCACCT-3') and human β-ACTIN cDNA (5'-GTG GGG CGC CCC AGG CAC CA-3' and 5'-CTC CTT AAT GTC ACG CAC GAT TTC-3'). The following PCR conditions were used: one cycle at 95 °C for 4 min; 35 cycles at 95 °C for 30 s, 58 °C for 30 s, and 72 °C for 50 s; and one cycle at 72 °C for 7 min. Cultured mouse lung SCC KAL^LU^ cells and human lung SCC cell lines H520 and SW900 were washed twice with PBS. Total RNA was isolated from cells using TRIzol (Invitrogen), precipitated, and reverse-transcribed (Applied Biosystems). Genes of interest were subsequently examined with greater sensitivity using RT-PCR with the TaqMan Universal PCR Master Mix and the ABI Prism 7300 Detection System (TaqMan; Applied Biosystems), according to the manufacturer’s instructions. Primer sets for mice included TNF (Mm00443260_g1), Twist1 (Mm04208233_g1), mouse UBCH10 (Mm00835439_g1), Bcl-2 (Mm00477631_m1), mouse Myc (Mm00487804_m1), mouse Sox2 (Mm03053810_s1), mouse TNFR1 (Mm00441883_g1), and human TNFR1 (Hs01042313_m1), all of which were purchased from Applied Biosystems. Gene expression was normalized to the level of the β-actin housekeeping gene. Data were analyzed using the ddCt method [48] and were expressed as a fold change in mRNA expression relative to control values.

### Cell proliferation assay

Mouse lung tumor cells (1 × 10^5^ /per well) were seeded into 6 well-plates in DMEM with 5% bovine calf serum. The numbers of the treated cells were counted 2 days after the cells were transfected with a control vector or HA-IKKα plasmid. Data represent the mean ± S.D. of three independent experiments. For the MTT assay to examine cell proliferation, the cells were plated in 96-well culture plate (2000 cells/200 µL/well) and incubated in a CO2 incubator; 20 µL of MTT (5 mg/mL, Sigma-Aldrich, M5655) was added to each well of a single plate and the cells incubated for 4 h at 37 °C. Medium was removed, and the formazan crystals formed by the cells were dissolved using 200 µL of DMSO for 30 min. The absorbance was read at 570 nm.

### siRNA transfection and cDNA transfection

All siRNAs were purchased from OriGene (Trilencer-27 siRNA), including *Tnfrsf1a* (mouse, TG517049), *Ube2c* (mouse, SR404321), *TNFRSF1A* (human: SR304883, SR517049; mouse: SR414807), *Tnf* (mouse, SR406508), and HPRT (positive-control siRNA duplex, SR30003). Transfection with Lipofectamine RNAiMAX was performed in accordance with the manufacturer’s instructions. The target protein expression was analyzed 48 h after transduction. Ube2c cDNA plasmid (MR223758L4V), which encodes UBCH10, was purchased from OriGene Biotechnology Research (Rockville).

### Chromatin immunoprecipitation (ChIP) assay

Cells were crosslinked with 1% formaldehyde and were lysed and sonicated on ice to generate DNA fragments with an average length of 200–800 bp. After preclearing, 1% of each sample was saved as an input fraction. Immunoprecipitation was performed using antibodies specifically recognizing IKKα (M-280, Santa Cruz Biotechnology), p65 and c-Rel. DNA was eluted and purified from complexes, followed by PCR amplification of the target promoters or genomic loci using primers for mouse *Ube2c* (*p-500*: 5'-CGG TCC TTG GAC CCT TTA AT-3' and 5'-TGC TAG GTC CTC CCC AGT AA-3'; *p-1000*: 5'-CGC TGT CTT CAG ACA CTC CA-3' and 5'-GAG ATG GCT CAG CGG TTA AG-3'; *p-1500*: 5'-ATA CAC CCT GGC TGA CCT TG-3' and 5'-GAA GGT GGT AAC AGG CAG GA-3'), human *UBE2C* (*p-500*: 5'-GTG GGC AAA AGG TGA GTG AT-3' and 5'-GAG CTC CTG GTG TGT TCT CC-3'; *p-1000*: 5'-GAT TAC GGA CGT GAG CCA CT-3' and 5'-GCG AGG GGA AAA ACT AAA GG-3'; *p-1500*: 5'-TAA GAC CAA CCT GGG AGC AC-3' and 5'-TGG GAG CAA TAA AAG CCA AC-3'), and mouse *Tnfrsf1a* (*p-500*: 5'-TGG CGT GAG TGA CTT TAG GT-3' and 5'-CCA CCC CAA GAA CCA ACA AG-3'). Also, the immunoprecipitation was performed using anti-H3K27ac antibody (ab4729, Abcam) in KAL^LU-^ and KAL^LU-^ cells. Then, DNA was eluted and purified from complexes, followed by PCR amplification of mouse *Tnfr1* promoters with its primer including forward: 5'-TGG CGT GAG TGA CTT TAG GT-3' and Reverse: 5'-CCA CCC CAA GAA CCA ACA AG-3'.

### Subcutaneous injection for tumor growth and metastasis studies

Approximately 2 × 10^5^ mouse lung SCC cells KAL^LU+^ cells (with or without si-RNA treatment) and KAL^LU-^ cells were injected subcutaneously into athymic NCr-nu/nu mice. Following the cell injection, the tumor growth was monitored with caliper measurements. At day 18 after cell injection, animals were euthanized when tumors reached the maximum size allowed by the guidelines of the Institutional Animal Care and Use Committee (IACUC) of the National Institutes of Health for all animal experiments. To study metastasis, the tumors derived from nude mice were digested with 0.05% trypsin solution at 37 °C for 20 min to make a single cell solution. Isolated cells were cultured for 7 days and then subcutaneously injected into nude mice. At day 18 after cell injection, animals were euthanized and the metastases in the liver and lungs were examined.

### Analyses for cancer patient survival

OncoLnc and Kaplan–Meier plotter were used to analyze the correlation between gene expression and patient survival rates. OncoLnc contains the survival data of 8,647 patients representing 21 cancer types, along with RNA-seq expression data of numerous genes performed by TCGA. The association of survival with a single gene was directly performed on the OncoLnc database [49]. For double gene analysis, raw data for each gene, including patient ID, survival days, status, and gene expression levels, were downloaded from OncoLnc and combined to compare the survival rate between double-high and double-low gene expression groups. The Kaplan–Meier plotter can be used to evaluate the effect of 54,675 genes on patient survival using 10,461 cancer samples. The “Use multiple gene” function was applied to analyze the correlation between double gene expression and patient survival [50]. The log-rank *p*-value and hazard ratio with 95% confidence intervals were determined.

## Supplementary information

Supplemental Figures

Original Data File

checklist

## Data Availability

The data supporting the present study are available from the corresponding author upon reasonable request.
