# Supplementary material for: A TNFR1–UBCH10 axis drives lung squamous cell carcinoma dedifferentiation and metastasis through a cell-autonomous signaling loop
Source: Cell Death Dis. 2022 Oct 21;13(10):885. doi: 10.1038/s41419-022-05308-4 (PMC9587052; doi:10.1038/s41419-022-05308-4)
Supplement: Supplementary file 1 — Supplemental Figures [file 41419_2022_5308_MOESM1_ESM.pdf]

A TNFR1–UBCH10 axis drives lung squamous cell carcinoma dedifferentiation and metastasis through a cell-autonomous signaling loop

Zuoxiang Xiao, Gongping Shi, Sichuan Xi, Amit Kumar Singh, Jami Willette-Brown, Xin Li, Feng Zhu, Ling Su, Xiaolin Wu, David S. Schrump, and Yinling Hu

Supplemental Figures

Figure S1

Figure S2

Figure S3

Figure S4

Figure S5

Figure S1

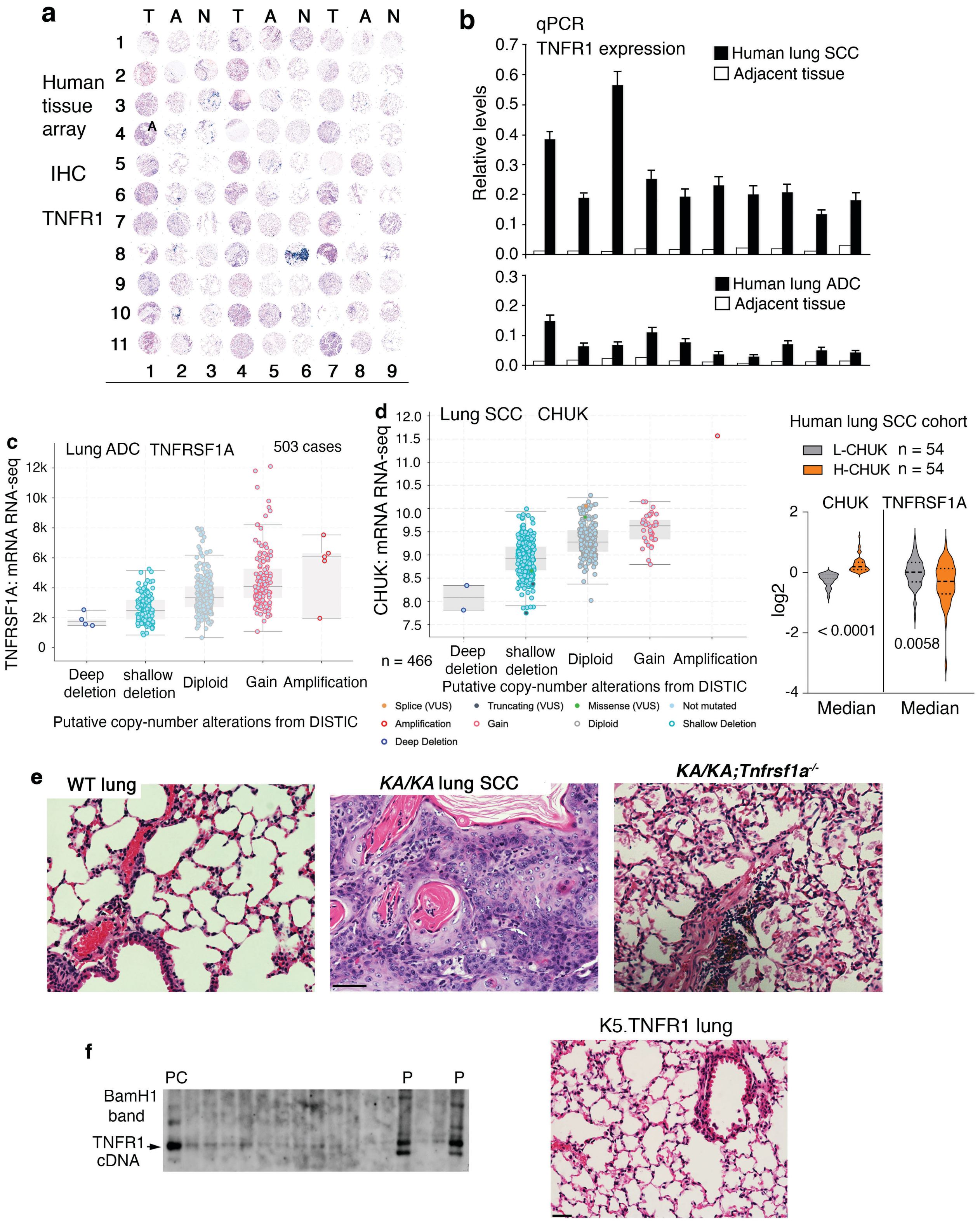

a
T A N T A N T A N
Human tissue array
IHC
TNFR1
b
qPCR
TNFR1 expression
Human lung SCC
Adjacent tissue
Relative levels
Human lung ADC
Adjacent tissue
c
Lung ADC TNFRSF1A
503 cases
TNFRSF1A: mRNA RNA-seq
Deep deletion
shallow deletion
Diploid
Gain
Amplification
Putative copy-number alterations from DISTIC
d
Lung SCC CHUK
CHUK: mRNA RNA-seq
n = 466
Deep deletion
shallow deletion
Diploid
Gain
Amplification
Putative copy-number alterations from DISTIC
Splice (VUS)
Truncating (VUS)
Missense (VUS)
Not mutated
Amplification
Gain
Diploid
Shallow Deletion
Deep Deletion
Human lung SCC cohort
L-CHUK n = 54
H-CHUK n = 54
CHUK
TNFRSF1A
log2
< 0.0001
0.0058
Median
Median
e
WT lung
KA/KA lung SCC
KA/KA;Tnfrsf1a-/-
f
PC
P
P
BamH1 band
TNFR1 cDNA
K5.TNFR1 lung

Fig. S1: Overexpression of TNFRSF1A in human lung SCC.

a Lung tissue array (BC04118, Biomax) stained with an anti-TNFR1 antibody. T, tumor; A, tissue adjacent to SCCs; N, normal lung tissue.

b RT-PCR analysis showing TNFR1 levels in human lung SCCs and their adjacent tissues.

c RNA-seq analyses showing *TNFRSF1A* expression in 507 human lung ADCs (TCGA, PanCancer, Atlas, cBioPortal).

d Left: RNA-seq analyses showing *CHUK* expression in 466 human lung SCCs (TCGA, PanCancer, Atlas, cBioPortal). Right: Correlation of high CHUK (H-CHUK) and low (L-CHUK), using median analysis to divide CHUK expression groups, with TNFRSF1A expression. Data were obtained from CPTAC, Cell 2021, cBioPortal. P values and sample numbers are indicated in the panel, Student's *t*-test.

e Hematoxylin and eosin (H&E) stained WT lung, *KA/KA* SCC, and *KA/KA;Tnfr1*$^{-/-}$ lung. Scale bar: 50 μM.

f Left: Southern blot identifies the mouse TNFR1 cDNA in Tg-K5.TNFR1 transgenic mice, as digested with BamH1 enzyme and probed with mouse TNFR1 cDNA. The TNFR1 cDNA indicated by an arrow was used as a positive control. Right: H&E-stained lung section of 2-month-old Tg-K5.TNFR1 mouse. Scale bar: 30 μM.

Figure S2

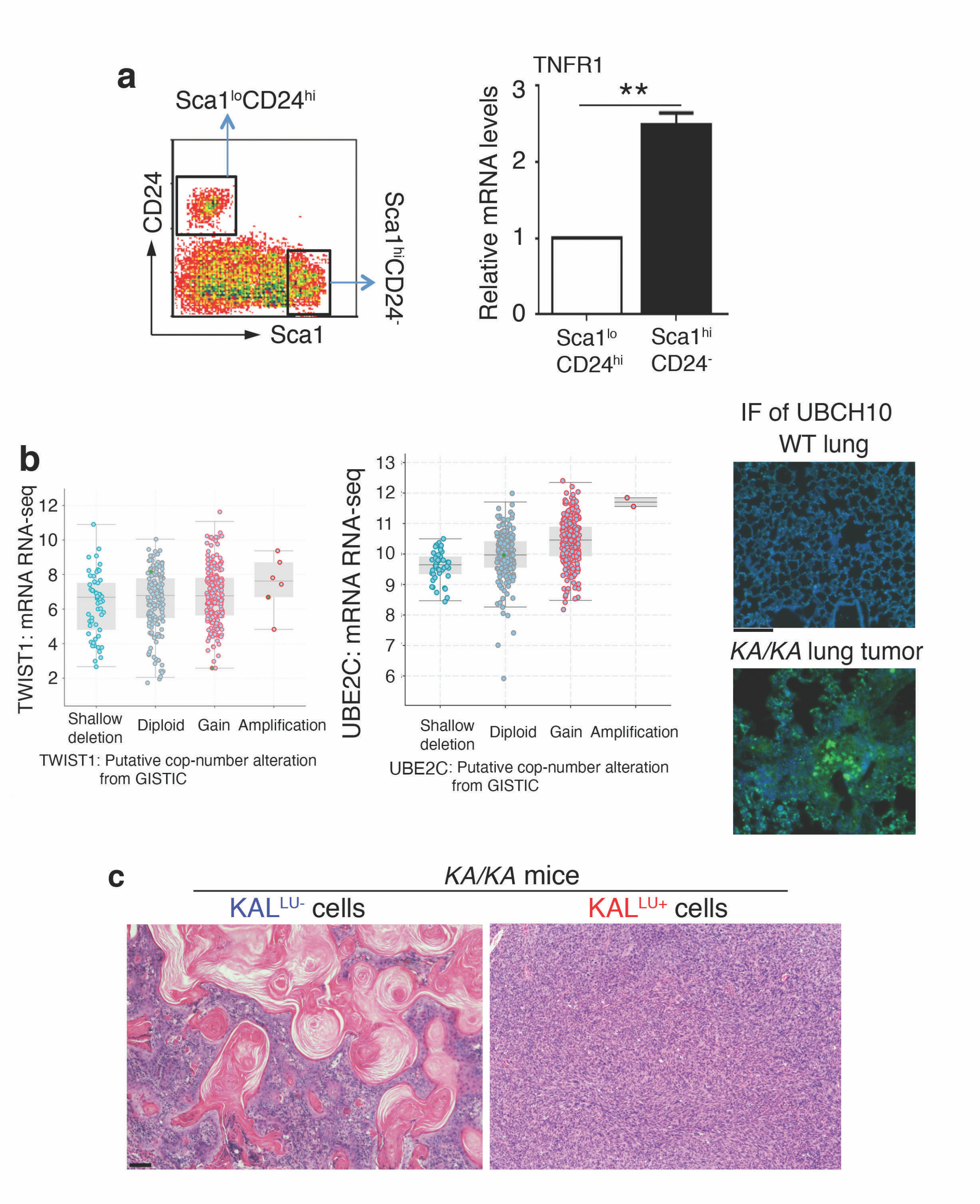
a
Sca1loCD24hi
CD24
Sca1
Sca1hiCD24-
TNFR1
**
Relative mRNA levels
0
1
2
3
Sca1lo CD24hi
Sca1hi CD24-
b
TWIST1: mRNA RNA-seq
Shallow deletion
Diploid
Gain
Amplification
TWIST1: Putative cop-number alteration from GISTIC
UBE2C: mRNA RNA-seq
UBE2C: Putative cop-number alteration from GISTIC
IF of UBCH10
WT lung
KA/KA lung tumor
c
KA/KA mice
KALLU- cells
KALLU+ cells

Fig. S2: Elevated TNFR1 expression is associated with stemness and dedifferentiated spindle cell carcinoma.

a KAL^{LU} cells were cultured for several generations. Further isolation of the KAL^{LU+} cells with Sca1 and CD24 markers (left panel), and examination of TNFR1 expression levels by RT-PCR (right panel). Mean ± SD (three samples per group); $**P < 0.01$; Student's *t*-test

b RNA-seq analyses showing TWIST1 (left) and UBE2C (middle) levels in a human lung SCC cohort containing 466 SCCs (TCGA, PanCancer, Atlas, cBioPortal). Immunofluorescent staining shows UBCH10 levels in lung SCC from *KA/KA* mice and WT lung (right). Green, positive for UBCH10; blue, DAPI nuclear staining. Scale bar: 40 μM.

c H&E-stained tumor sections generated by KAL^{LU-} and KAL^{LU+} cell injections (see Figure 2f). Scale bar: 40 μM.

Figure S3

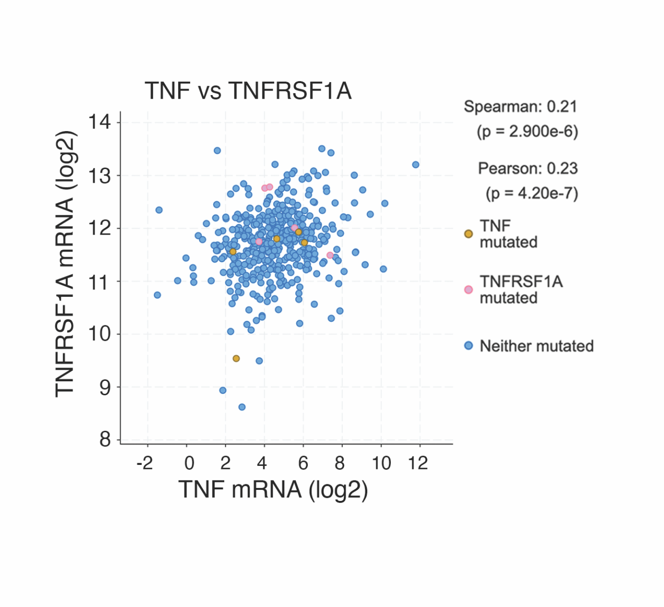

Fig. S3: Correlation between TNFR1 and TNF in human lung SCC.

Correlation between TNFRSF1A and TNF expression in a human lung SCC cohort containing 466 SCCs (TCGA, PanCancer Atlas, cBioPortal).

Figure S4

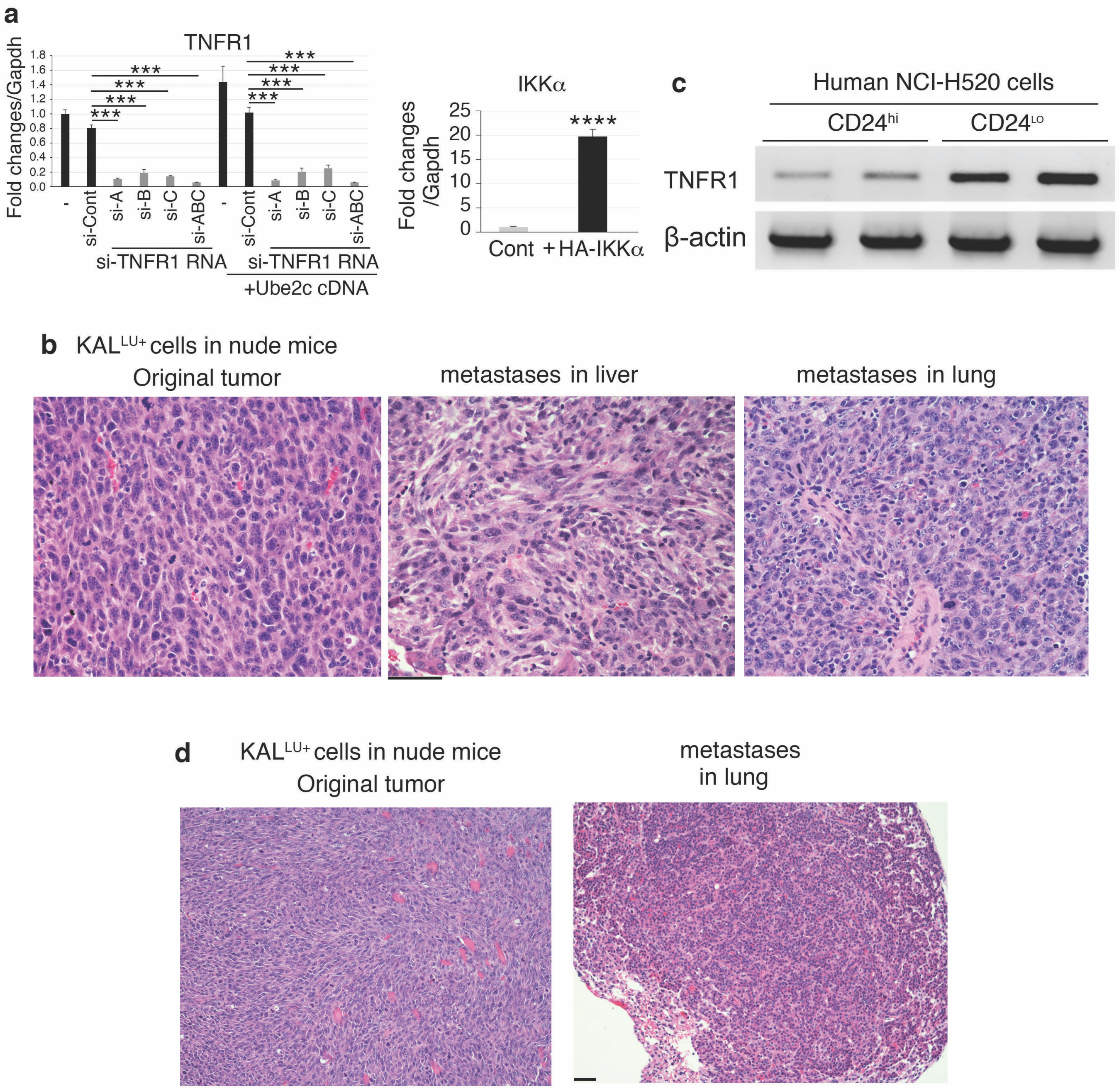
a
TNFR1
Fold changes/Gapdh
1.8
1.6
1.4
1.2
1.0
0.8
0.6
0.4
0.2
0.0
***
-
si-Cont
si-A
si-B
si-C
si-ABC
si-TNFR1 RNA
+Ube2c cDNA
IKKα
Fold changes /Gapdh
25
20
15
10
5
0
****
Cont
+HA-IKKα
c
Human NCI-H520 cells
CD24hi
CD24LO
TNFR1
β-actin
b
KALLU+ cells in nude mice
Original tumor
metastases in liver
metastases in lung
d
KALLU+ cells in nude mice
Original tumor
metastases
in lung

Fig. S4: Elevated TNFR1 expression is associated with tumor metastasis in mice and stemness in human SCC cells.

a Left: RT-PCR detects TNFR1 expression in KAL^{LU+} (-), si-TNFR1 RNA-treated KAL^{LU+} cells (si-TNFR1 RNA including si-A, si-B, si-C, and si-ABC), and si-TNFR1 RNA-treated KAL^{LU+} cells with reintroduced Ube2c/UBCH10 cDNA. Mean ± SD (four samples per group); ****P* < 0.001; Student's *t*-test. Right: RT-PCR detects IKKα expression in KAL^{LU+} cells with introduced vector DNA (Cont) and HA-IKKα cDNA). Mean ± SD (three samples per group); *****P* < 0.0001; Student's *t*-test.

b H&E-stained metastases in the liver and lung and the original lung tumor derived from KAL^{LU+} cell (5×10^5 cells per mouse) injection. Scale bar: 40 μM.

c RT-PCR showing TNFR1 expression levels in isolated $CD24^{hi}$ and $CD24^{lo}$ cells from H520 cells. These cells were CD44-positive. β-actin is a mRNA loading control.

d H&E-stained original tumors derived from human NCI-H520 cell injection and metastases in lungs (see Fig. 4I). Scale bar: 40 μM.

Figure S5

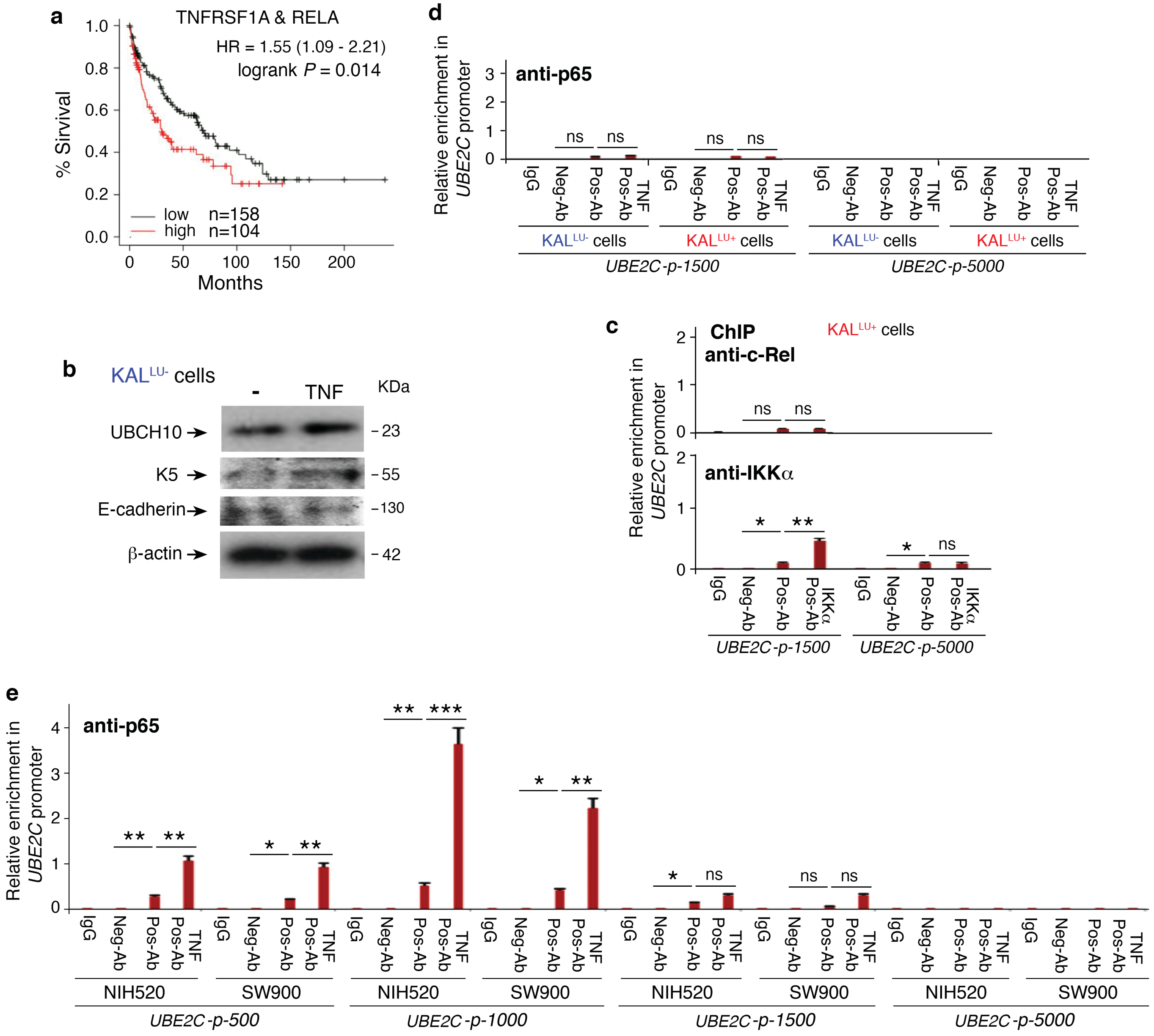
a
TNFRSF1A & RELA
HR = 1.55 (1.09 - 2.21)
logrank P = 0.014
% Sirvival
low n=158
high n=104
Months
b
KALLU- cells
-
TNF
KDa
UBCH10
K5
E-cadherin
β-actin
23
55
130
42
c
ChIP
anti-c-Rel
KALLU+ cells
anti-IKKα
Relative enrichment in UBE2C promoter
IgG
Neg-Ab
Pos-Ab
IKKα Pos-Ab
UBE2C-p-1500
UBE2C-p-5000
ns
*
**
d
anti-p65
Relative enrichment in UBE2C promoter
IgG
Neg-Ab
Pos-Ab
TNF Pos-Ab
KALLU- cells
KALLU+ cells
UBE2C-p-1500
UBE2C-p-5000
e
anti-p65
Relative enrichment in UBE2C promoter
NIH520
SW900
UBE2C-p-500
UBE2C-p-1000
UBE2C-p-1500
UBE2C-p-5000
***

Fig. S5: Regulation of expression of the *UBE2C* gene at a transcriptional level.

a Survival curves of human lung SCC expressing double-high TNFRSF1A and RELA levels compared to low TNFRSF1A levels. The data were obtained from the Kaplan-Meier plotter.

b Immunoblotting showing indicated protein levels in KAL^{LU-} cells following TNF (10 ng/mL) for 60 minutes. β-actin, a protein loading control.

c ChIP assay shows the relative enrichments of c-Rel and IKKα on the *Ube2c* promoter (p), with antibodies against c-Rel and IKKα for immunoprecipitation and with PCR primers for the *Ube2c* promoter at regions upstream of -1,500 bp and -5,000 bp in KAL^{LU+} cells. The relative enrichments were compared to input levels. anti- or Ab, antibody; Neg-Ab, negative-control antibody (Ig); Pos-Ab, positive experimental antibody; +IKKα, overexpressed HA-IKKα in KAL^{LU+} cells. Mean ± SD (three samples per group); *$P < 0.05$; **$P < 0.01$; ns, not significant; Student's *t*-test.

d ChIP assay shows the relative enrichment of p65 with the *Ube2c* promoter (p), with anti-p65 antibody for immunoprecipitation, and with PCR primers for the *Ube2c* promoter at regions upstream of -1,500 bp and -5,000 bp in KAL^{LU-} and KAL^{LU+} cells. The relative enrichment was compared to input levels. anti- or Ab, antibody; Neg-Ab, negative-control antibody (Ig); Pos-Ab, positive experimental antibody; TNF, TNF (10 ng/mL) treatment for 45 minutes. Mean ± SD (three samples per group); ns, not significant; Student's *t*-test.

e ChIP assay shows the enrichment of p65 with the *Ube2c* promoter (p), with anti-p65 antibody for immunoprecipitation, and with PCR primers for the *Ube2c* promoter at regions upstream of -500 bp, -1,000 bp, -1,500 bp, and -5,000 bp in human H520 and SW900 cells. The relative enrichments were compared to input levels. anti-, antibody; Neg-Ab, negative control antibody; Pos-Ab, positive experimental antibody; TNF, TNF (10 ng/mL) treatment for 45 minutes. Mean ± SD (three samples per group); *$P < 0.05$; **$P < 0.01$; ***$P < 0.001$; ns, not significant; Student's *t*-test.
