# Supplementary material for: A TNFR1–UBCH10 axis drives lung squamous cell carcinoma dedifferentiation and metastasis through a cell-autonomous signaling loop
Source: Cell Death Dis. 2022 Oct 21;13(10):885. doi: 10.1038/s41419-022-05308-4 (PMC9587052; doi:10.1038/s41419-022-05308-4)
Supplement: Supplementary file 2 — Original Data File [file 41419_2022_5308_MOESM2_ESM.pdf]

Supplemental Figures for original western blots

Figure 1

Figure 1b:

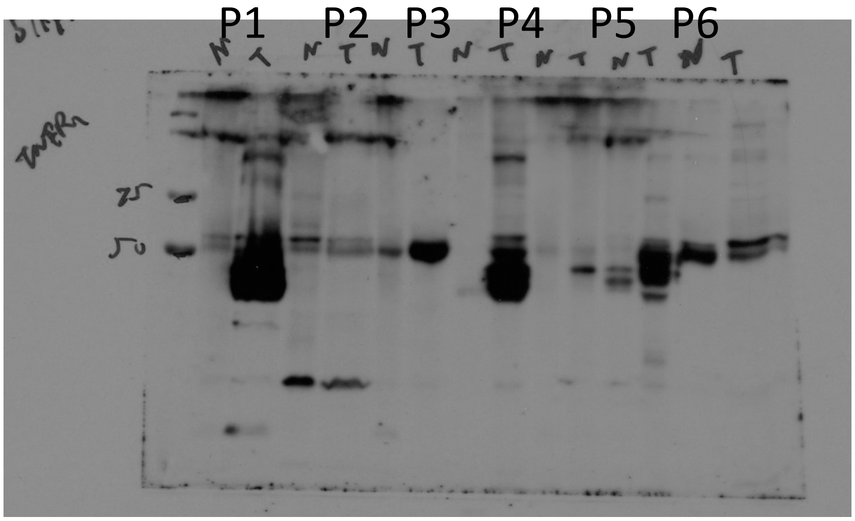

TNFR1
TNFR1 levels
N (Ad), Adjacent tissue
T, Tumor-human lung SCC
P1-7, lung SCC patients

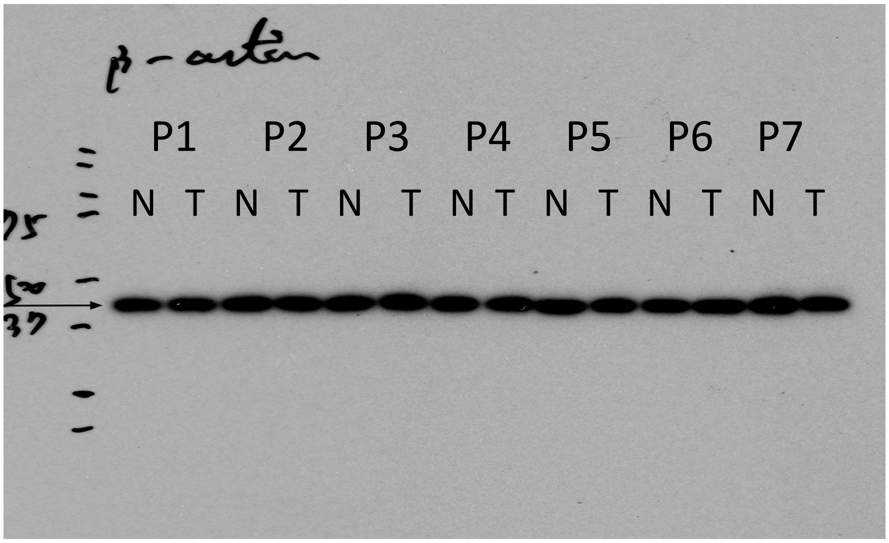

β–actin
β-actin levels indicated by an arrow.
N (Ad), Adjacent tissue
T, Tumor-human lung SCC
P1-7, lung SCC patients

Figure 1e:

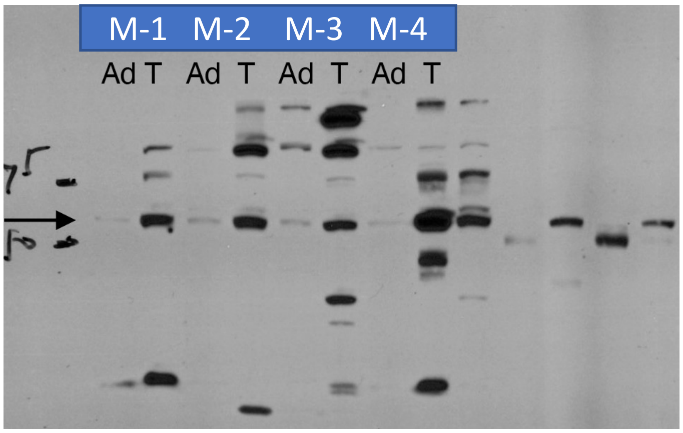

TNFR1
TNFR1 levels indicated by an arrow in adjacent lung tissues (Ad) and lung SCCs (T) from KA/KA mice (M-1 to M4).

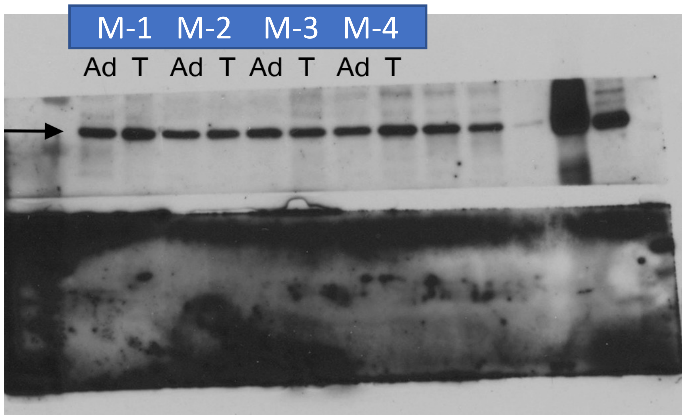

β-actin
β-actin levels indicated by an arrow in adjacent lung tissues (Ad) and lung SCCs (T) from KA/KA mice (M1 to M4)

Figure 2

Figure 2a:

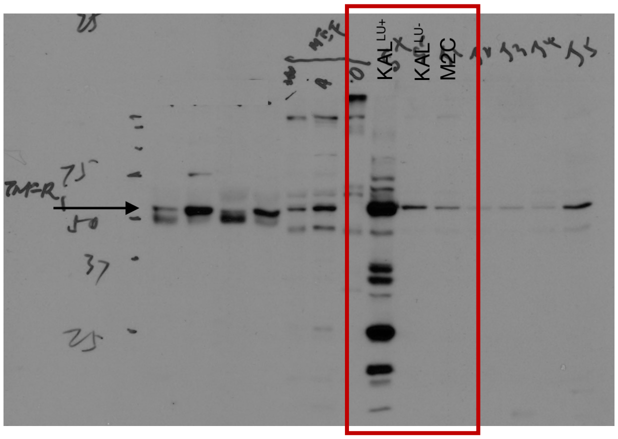

TNFR1
TNFR1 levels indicated by an arrow in KAL^{LU+}, KAL^{LU-}, and M2C cells indicated within a red box.

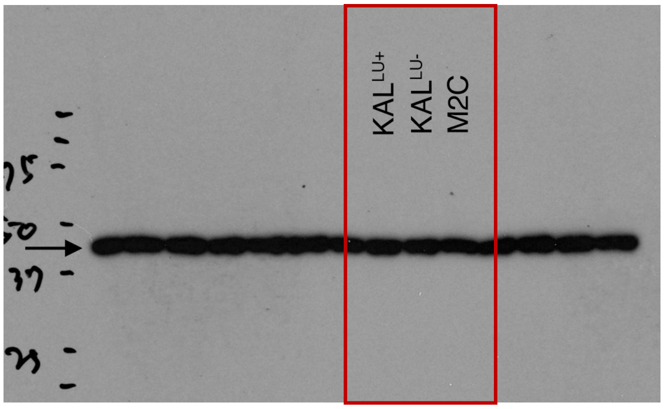

β-actin
β-actin levels indicated by an arrow in KAL^{LU+}, KAL^{LU-}, and M2C cells.

Figure 2c:

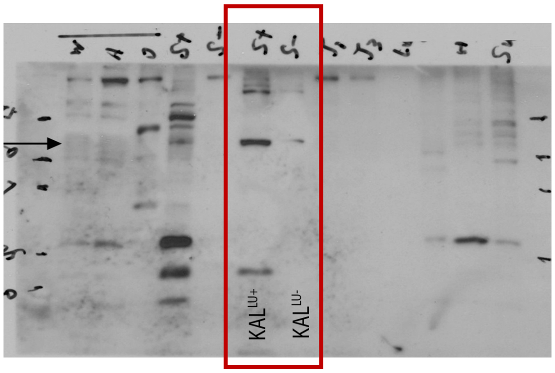

TNFR1
TNFR1 levels indicated by an arrow in KAL^{LU+} and KAL^{LU-} cells within a red box.

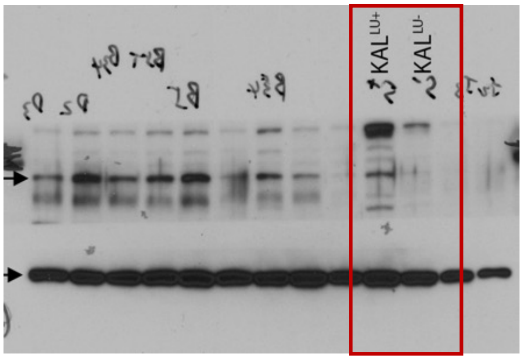

c-Myc and β-actin
c-Myc levels indicated by an upper arrow and **β-actin** levels indicated by a low arrow in KAL^{LU+} and KAL^{LU-} cells within a red box.

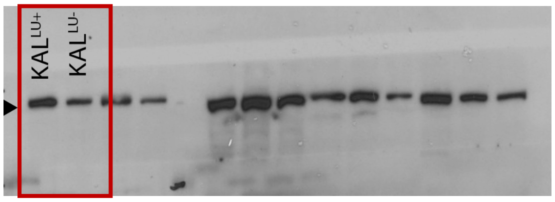

Sox2
Sox2 levels indicated by an arrow in in KAL^{LU+} and KAL^{LU-} cells within a red box.

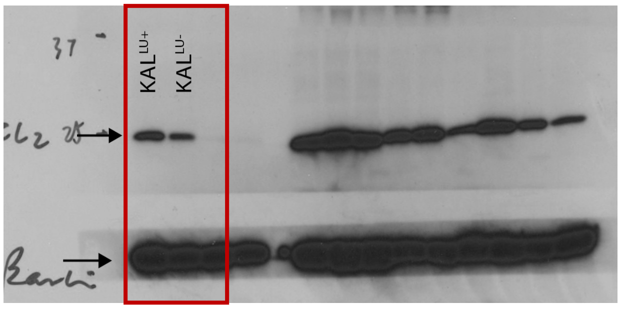

Bcl2
Bcl2 levels indicated by an arrow in KAL^{LU+} and KAL^{LU-} cells within a red box.

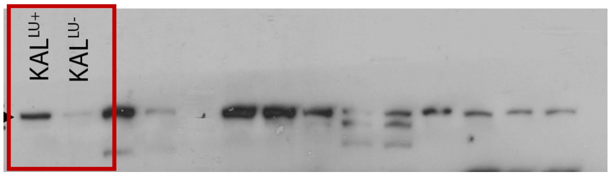

Twist1
Twist1 levels indicated by an arrow in KAL^{LU+} and KAL^{LU-} cells within a red box.

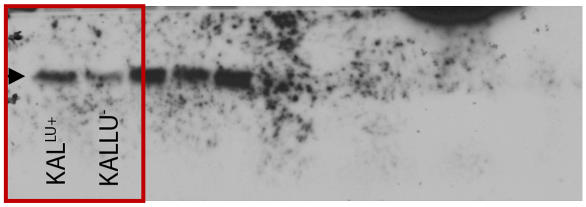

UBCH10
UBCH10 levels indicated by an arrow in KAL^{LU+} and KAL^{LU-} cells within a red box.

Figure 3

Figure 3a:

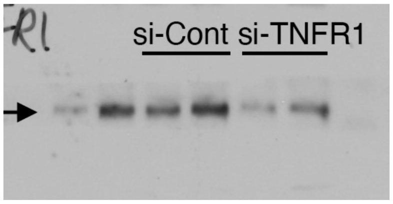

TNFR1
TNFR1 levels indicated by an arrow in KAL^{LU+} cells treated with si-Control or si-TNFR1 RNA.

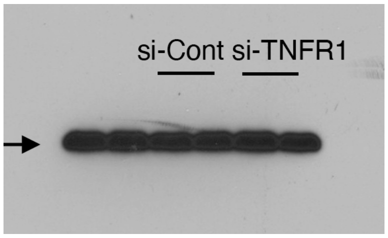

β-actin
β-actin levels indicated by an arrow in KAL^{LU+} cells treated with si-Control or si-TNFR1 RNA.

Figure 3c:

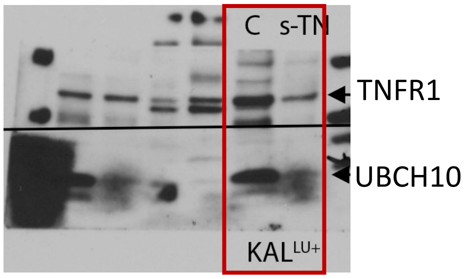

TNFR1 and UBCH10
TNFR1 (top) and **UBCH10** (low) levels from two blots indicated by arrows in KAL^{LU+} cells treated with and without si-TNFR1 RNA within a red box. s-TN, si-TNFR1 RNA; C, si-RNA control.

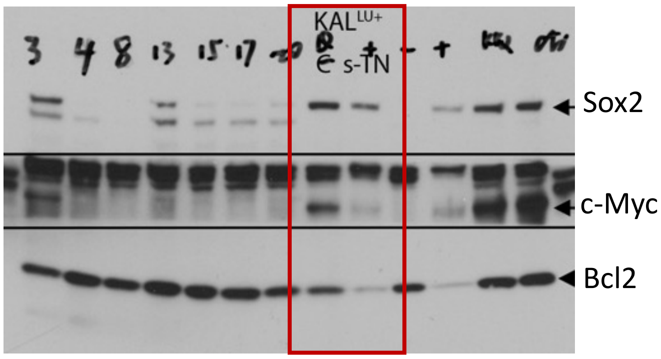

Sox2, c-Myc, and Bcl2
Sox2, **c-Myc**, and **Bcl2** levels from three blots indicated by arrows in KALLU+ cells treated with and without si-TNFR1 RNA within a red box. s-TN, si-TNFR1 RNA; C, si-RNA control.

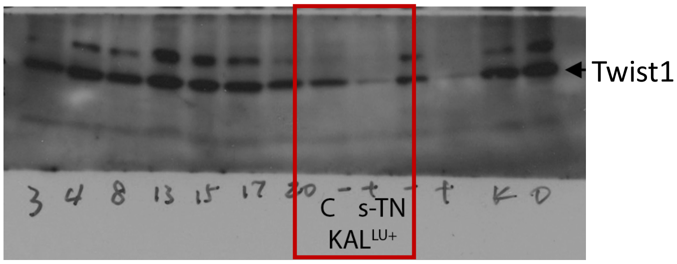

Twist 1
Twist 1 levels indicated by an arrow in KALLU+ cells treated with and without si-TNFR1 RNA within a red box. s-TN, si-TNFR1 RNA; C, si-RNA control.

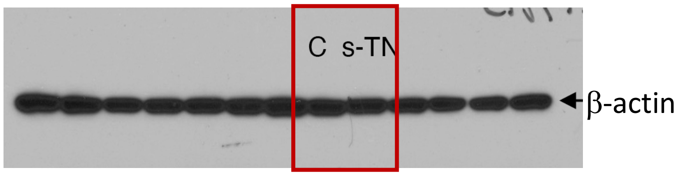

β-actin
β-actin levels indicated by an arrow in KALLU+ cells treated with and without si-TNFR1 RNA within a red box. s-TN, si-TNFR1 RNA; C, si-RNA control.

Figure 3h:

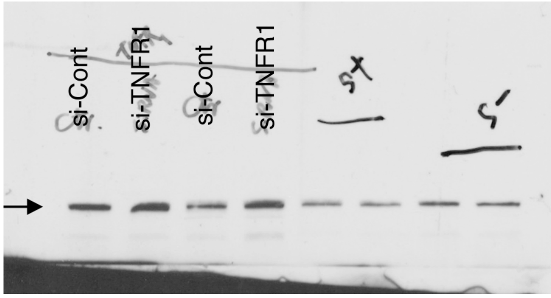

Keratin 5 (K5) levels indicated by an arrow in KAL^{LU+} cells treated with si-control RNA (si-Cont) or si-TNFR1 RNA.

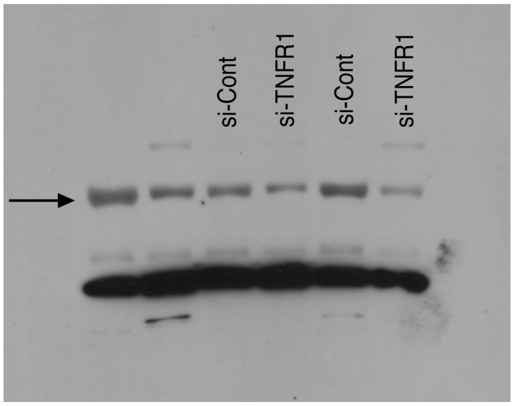

TNFR1

TNFR1 levels indicated by an arrow in KAL^{LU+} cells treated with si-RNA RNA or si-TNFR1 RNA.

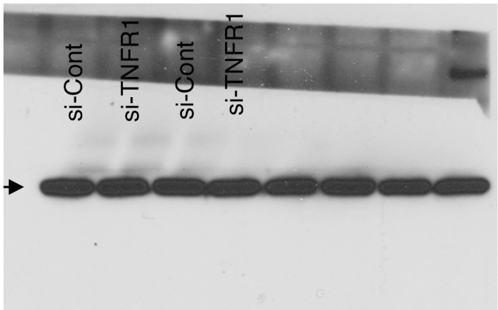

β-actin

β-actin levels indicated by an arrow in KAL^{LU+} cells treated with si-control RNA (si-Cont) or si-TNFR1 RNA.

Figure 4

Figure 4b:

UBCH10

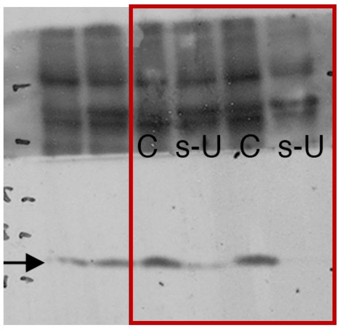

UBCH10

UBCH10 levels indicated by an arrow in KAL^{LU+} cells treated with si-control RNA (si-Cont) or si-UBE2C RNA (s-U).

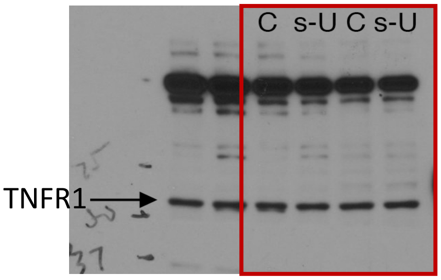

TNFR1

TNFR1 levels indicated by an arrow in KAL^{LU+} cells treated with si-control RNA (si-Cont) or si-UBE2C RNA (s-U).

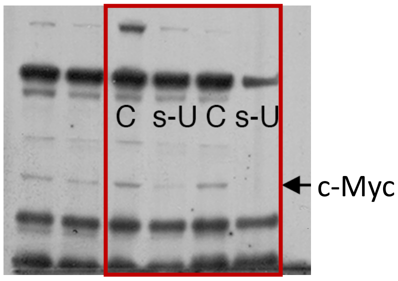

c-Myc

c-Myc levels indicated by an arrow in KAL^{LU+} cells treated with si-control RNA (si-Cont) or si-UBE2C RNA (s-U).

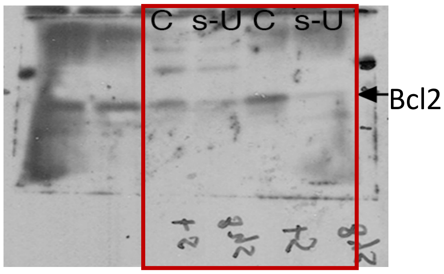

Bcl2

Bcl2 levels indicated by an arrow in KAL^{LU+} cells treated with si-control RNA (si-Cont) or si-UBE2C RNA (s-U).

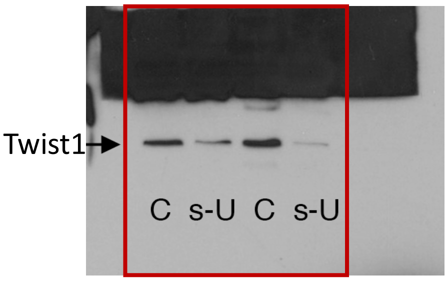

Twist1

Twist1 levels indicated by an arrow in KAL^{LU+} cells treated with si-control RNA (si-Cont) or si-UBE2C RNA (s-U).

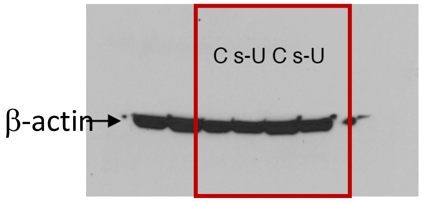

β-actin

β-actin levels indicated by an arrow in KAL^{LU+} cells treated with si-control RNA (si-Cont) or si-UBE2C RNA (s-U).

Figure 4b, right:

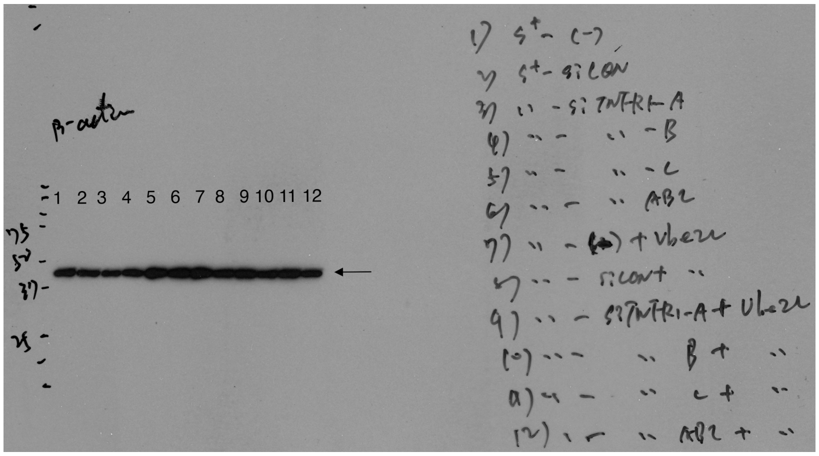

β-actin

β-actin levels indicated by an arrow in different KAL^{LU+} cells:

1: KAL^{LU+} cells only (-)

2: Si-control RNA

3-6: si-TNFR1 RNA A, B, C, and ABC

7: Ube2c/UBCH10 cDNA

8-12: si-TNFR1 RNA A, B, C, and ABC + Ube2c/UBCH10 cDNA

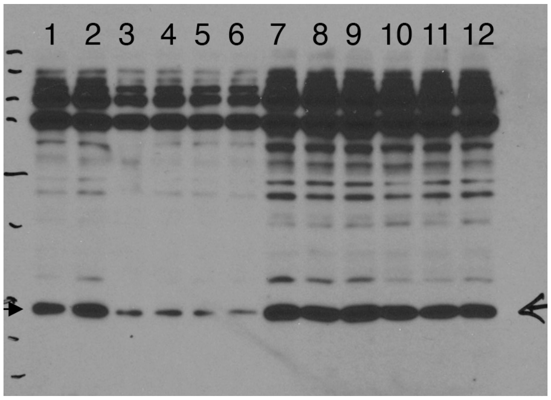

Twist1

Twist1 levels indicated by an arrow in different KAL^{LU+} cells:

1: KAL^{LU+} cells only (-)

2: Si-control RNA

3-6: si-TNFR1 RNA A, B, C, and ABC

7: Ube2c/UBCH10 cDNA

8-12: si-TNFR1 RNA A, B, C, and ABC + Ube2c/UBCH10 cDNA

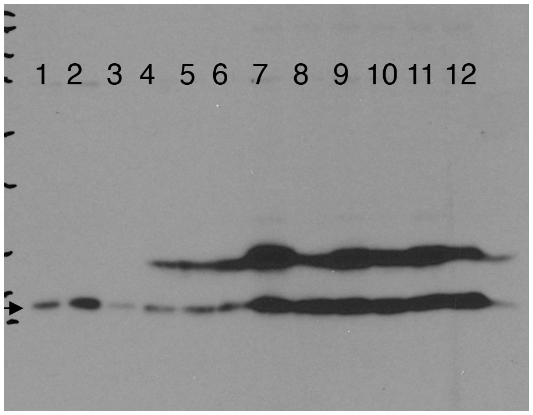

UBCH10

UBCH10 levels indicated by an arrow in different KAL^{LU+} cells:

1: KAL^{LU+} cells only (-)

2: Si-control RNA

3-6: si-TNFR1 RNA A, B, C, and ABC

7: Ube2c/UBCH10 cDNA

8-12: si-TNFR1 RNA A, B, C, and ABC + Ube2c/UBCH10 cDNA

Figure 4e:

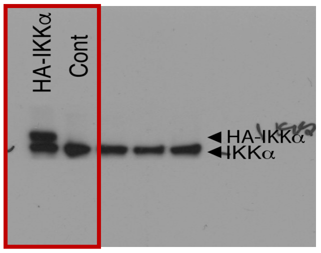

HA-IKKα and **IKKα**

HA-IKKα and **IKKα** indicated by arrows in KAL[LU+] cells with and without HA-IKKα. Cont, Control.

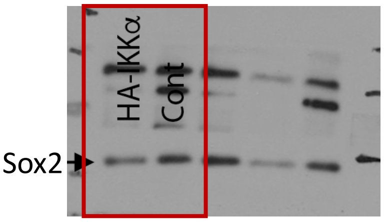

Sox2

Sox2 levels indicated by an arrow in KAL[LU+] cells with and without HA-IKKα expression. Cont, Control.

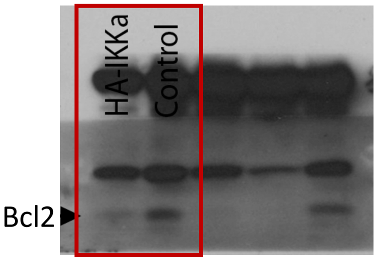

Bcl2

Bcl2 levels indicated by an arrow in KAL[LU+] cells with and without overexpressed HA-IKKα.

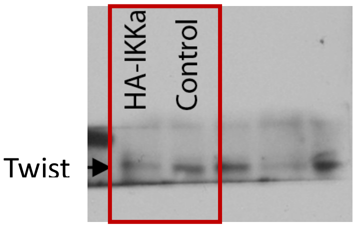

Twist1

Twist1 levels indicated by an arrow in KAL[LU+] cells with and without HA-IKKα.

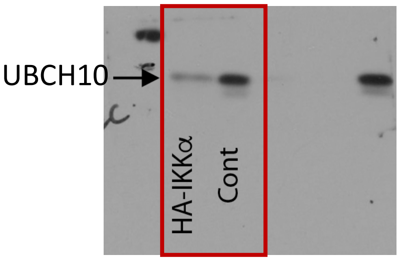

UBCH10

UBCH10 levels indicated an arrow in KAL[LU+] cells with and without HA-IKKα. Cont, Control.

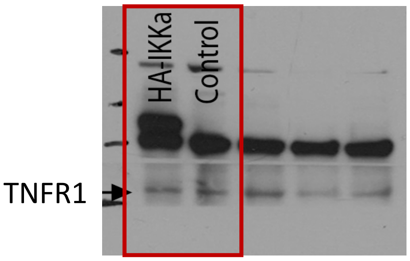

TNFR1
TNFR1levels indicated by an arrow in KAL^{LU+} cells with and without HA-IKKα.

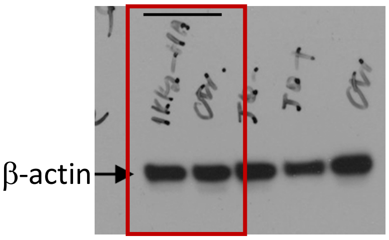

β-actin
β-actin levels indicated by an arrow in KAL^{LU+} cell with and without HA-IKKα.

Figure 5

Figure 5a:

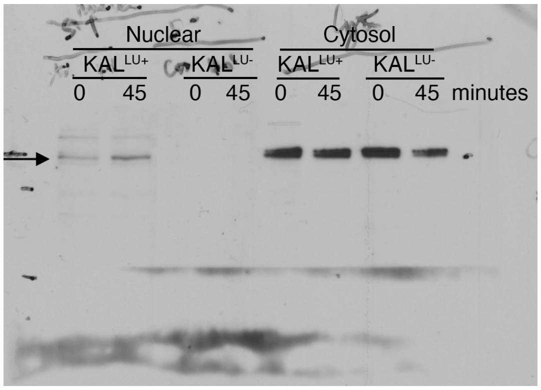

c-Rel
c-Rel nuclear and cytosol protein levels indicated by an arrow in KAL^{LU+} and KAL^{LU-} cells following TNF treatment at 0 and 45 minutes.

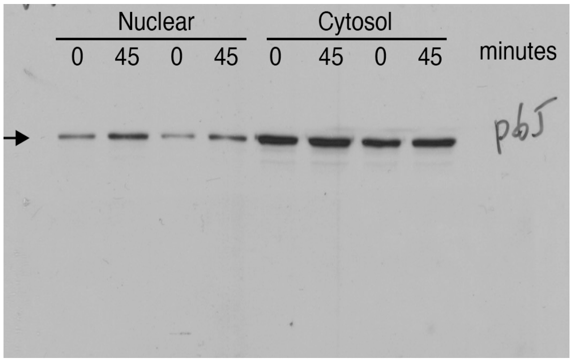

p65
p65 nuclear and cytosol protein levels indicated by an arrow in KAL^{LU+} and KAL^{LU-} cells following TNF treatment at 0 and 45 minutes.

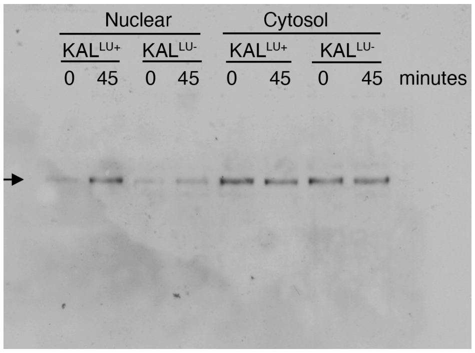

p50
p50 nuclear and cytosol protein levels indicated by an arrow in KAL^{LU+} and KAL^{LU-} cells following TNF treatment at 0 and 45 minutes.

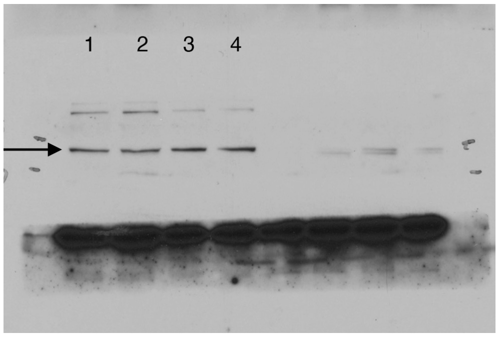

Lamin B1
Lamin B1 levels indicated by an arrow as a nuclear protein loading control from number 1 to 4 in Figure 5a.

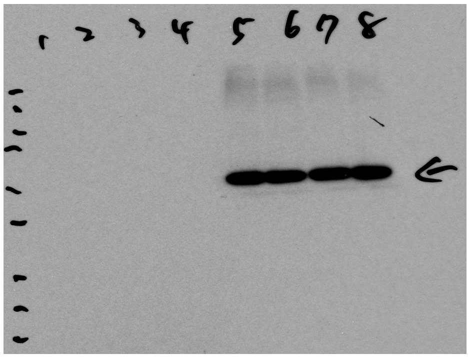

α-Tubulin
α-Tubulin cytosol protein levels indicated by an arrow, used as a cytosol protein loading control from number 5 to 8 in Figure 5a.

Figure 5c:

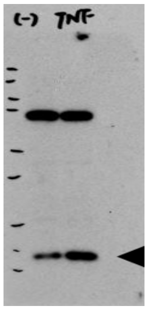

UBCH10
UBCH10 levels indicated by an arrow in KAL^{LU+} cells following TNF stimulation.

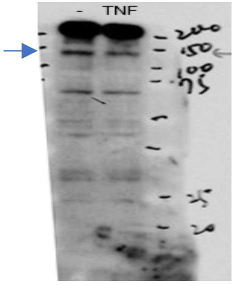

E-cadherin
E-cadherin levels indicated by an arrow in KAL^{LU+} cells following TNF stimulation.

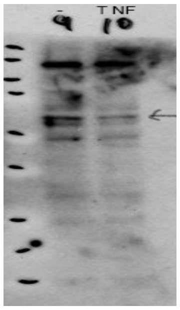

keratin 5 (**K5**)
K5 levels indicated by an arrow in KAL^{LU+} cells following TNF stimulation.

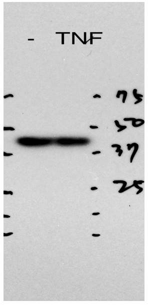

β-actin
β-actin levels in KAL^{LU+} cells following TNF stimulation.

Figure 5e:

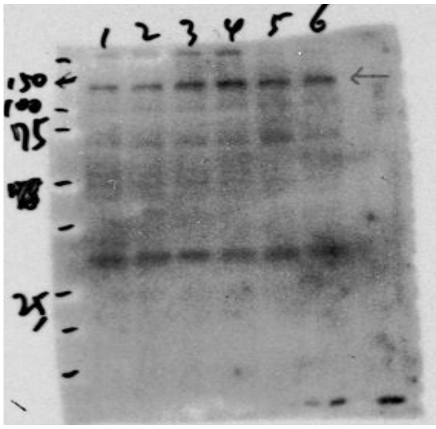

E-cadherin
E-cadherin levels indicated by an arrow in KAL^{LU+} cells following si-UBE2C RNA treatment:
1 un-treatment
2 si-control RNA treatment
3 si-UBE2C RNA: A
4 si-UBE2C RNA: B
5 si-UBE2C RNA: C
6 si-UBE2C RNA: A+B+C
UBE2C gene encoding UBCH10

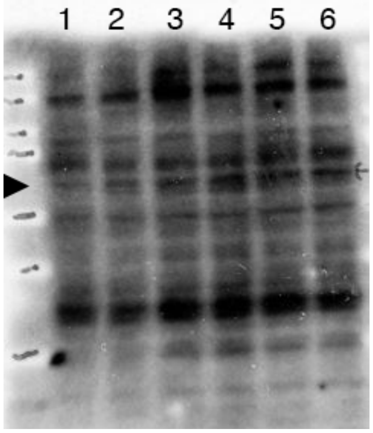

Keratin (**K5**)
K5 levels indicated by an arrow in KAL^{LU+} cells following si-UBE2C RNA treatment:
1 un-treatment
2 si-control RNA treatment
3 si-UBE2C RNA: A
4 si-UBE2C RNA: B
5 si-UBE2C RNA: C
6 si-UBE2C RNA: A+B+C
UBE2C gene encoding UBCH10

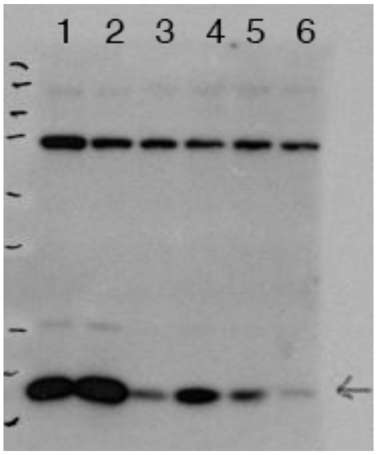

UBCH10
UBCH10 levels indicated by an arrow in KAL^{LU+} cells following si-UBE2C RNA treatment:
1 un-treatment
2 si-control RNA treatment
3 si-UBE2C RNA: A
4 si-UBE2C RNA: B
5 si-UBE2C RNA: C
6 si-UBE2C RNA: A+B+C
UBE2C gene encoding UBCH10

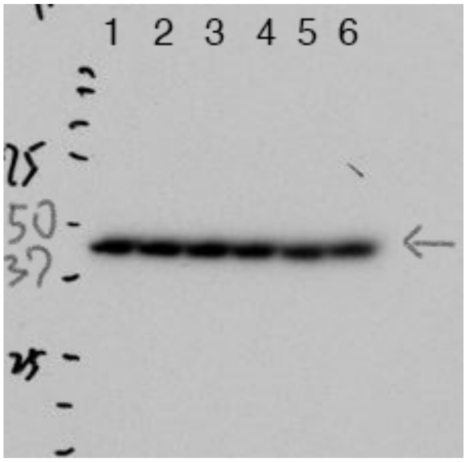

β-actin
β-actin levels indicated by an arrow in KAL^{LU+} cells following si-UBE2C RNA treatment:
1 un-treatment
2 si-control RNA treatment
3 si-UBE2C RNA: A
4 si-UBE2C RNA: B
5 si-UBE2C RNA: C
6 si-UBE2C RNA: A+B+C
UBE2C gene encoding UBCH10

Supplemental Figure 5

Figure S5b:

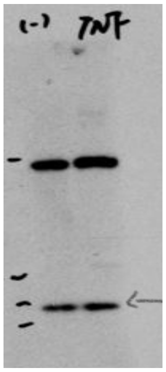

UBCH10
UBCH10 levels indicated by an arrow in KAL^{LU-} cells following TNF treatment.

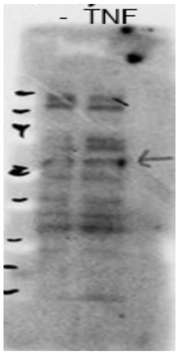

Keratin 5 (**K5**)
K5 levels indicated by an arrow in KAL^{LU-} cells following TNF treatment.

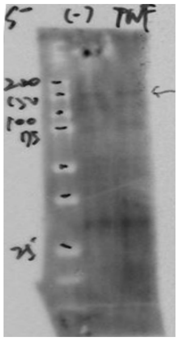

E-cadherin
E-cadherin levels indicated by an arrow in KAL^{LU-} cells following TNF treatment.

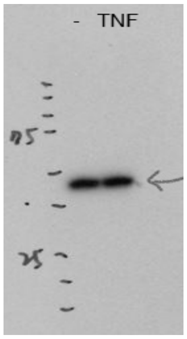

β-actin
β-actin levels indicated by an arrow in KAL^{LU-} cells following TNF treatment.
