# Supplementary material for: A TNFR1–UBCH10 axis drives lung squamous cell carcinoma dedifferentiation and metastasis through a cell-autonomous signaling loop
Source: Cell Death Dis. 2022 Oct 21;13(10):885. doi: 10.1038/s41419-022-05308-4 (PMC9587052; doi:10.1038/s41419-022-05308-4)
Supplement: Supplementary file 3 — checklist [file 41419_2022_5308_MOESM3_ESM.pdf]

Corresponding Author Name: Yinling Hu
Manuscript Number: CDDIS-22-1698

SPRINGER NATURE

Reporting Summary

Springer Nature wishes to improve the reproducibility of the work that we publish. This checklist is used to ensure good reporting standards and to improve the reproducibility. Please respond completely to all questions relevant to your manuscript. For more information, please read the journal's Guide to Authors.

☐ **Check here to confirm that the following information is available in the Material & Methods section:**

- **The exact sample size (*n*)** for each experimental group/condition, given as a number, not a range
- **A description of the sample collection** allowing the reader to understand whether the samples represent technical or biological replicates (including how many animals, litters, culture, etc.)
- **A statement of how many times the experiment shown was replicated in the laboratory**
- **Definitions of statistical methods and measures**: For small sample sizes (n<5) descriptive statistics are not appropriate, instead plot individual data points
  - Very common tests, such as *t*-test, simple χ^2 tests, Wilcoxon and Mann-Whitney tests, can be unambiguously identified by name only, but more complex techniques should be described in the methods section
  - Are tests one-sided or two-sided?
  - Are there adjustments for multiple comparisons?
  - **Statistical test results**, e.g., ***P* values**
  - Definition of **'center values'** as **median or mean**;
  - Definition of **error bars as s.d. or s.e.m. or c.i.**

Please ensure that the answers to the following questions are reported in the manuscript itself. We encourage you to include a specific subsection in the methods section for statistics, reagents and animal models. Below, provide the page number or section and paragraph number.

Statistics and general methods	Reported in section/paragraph or page #
1. How was the sample size chosen to ensure adequate power to detect a pre-specified effect size? (Give section/paragraph or page #)	Animal numbers in each group for each experiment are indicated in Fig. 1g and figure legends: line 621, 642, 653, 654, 657, 669, 678, and 680. The results of these experiments are statistically analyzed, and P values are indicated. Sample numbers in a human tissue array are
For animal studies, include a statement about sample size estimate even if no statistical methods were used.	All animal experiments were statistically analyzed and their results are statistically significant.
2. Describe inclusion/exclusion criteria if samples or animals were excluded from the analysis. Were the criteria pre-established? (Give section/paragraph or page #)	We did not exclude any numbers of all animal experiments in this manuscript.
3. If a method of randomization was used to determine how samples/animals were allocated to experimental groups and processed, describe it. (Give section/paragraph or page #)	We randomly picked up animals with right age and genotypes for each experiment (page 17).
For animal studies, include a statement about randomization even if no randomization was used.	A statement can be found in line 392.

4. If the investigator was blinded to the group allocation during the experiment and/or when assessing the outcome, state the extent of blinding. (Give section/paragraph or page #)	We used genetically modified animals that develop spontaneous lung SCCs or wild-type mice that received injected different tumor cell lines. Then we evaluated the tumor development. We did not treat these experimental animals with drugs. Thus, each experiment of these animal experiments contained two groups (see Q3 above: page 17).
For animal studies, include a statement about blinding even if no blinding was done.	A statement can be found in line 393.
5. For every figure, are statistical tests justified as appropriate?	Yes, please see Figure 1 to Figure 5 and Figure legends from page 26 to 32. All these results are appropriately justified and statistically analyzed. P values and their analyzing methods are indicated in these figure legends
Do the data meet the assumptions of the tests (e.g., normal distribution)?	Yes.
Is there an estimate of variation within each group of data?	All the data were statistically analyzed in each experiment including animal experiments, cell growth, and gene expression levels.
Is the variance similar between the groups that are being statistically compared? (Give section/paragraph or page #)	Yes. Comparison of two group in each experiment was indicated by drawing lines in figure 1 to 5 (Fig. 1a, c, d, g; Fig. 2d, e; Fig. 3b, d, e, i, j, k, l; Fig. 3a, c, g, i; Fig. 4d, e, f, g, i, k; Fig. 5d, f, g, h, i.

Reagents

Reported in section/paragraph or page #

6. Report the source of antibodies (vendor and catalog number)	Yes, we provided which companies I purchased antibodies and their catalogues numbers (line 413 to 419).
7. Identify the source of cell lines and report if they were recently authenticated (e.g., by STR profiling) and tested for mycoplasma contamination	We provided the reference for the cell lines we used for this manuscript line 130 (Ref. 3).

Animal Models

Reported in section/paragraph or page #

8. Report species, strain, sex and age of animals	We reported animal species, strains, sex and age of animals in this study in page 7 and page 24.
9. For experiments involving live vertebrates, include a statement of compliance with ethical regulations and identify the committee(s) approving the experiments.	All mouse experiments used for this manuscript are approved by the Institutional Animal Care and Use Committee (IACUC) and animal protocols include 11-051, 11-052, 14-051, 14-052, 20-051, and 20-052.

10. We recommend consulting the ARRIVE guidelines (*PLoS Biol.* **8**(6), e1000412,2010) to ensure that other relevant aspects of animal studies are adequately reported.

Human subjects

Reported in section/paragraph or page #

11. Identify the committee(s) approving the study protocol.	All human samples used in Fig. 1b and S1b were obtained The Thoracic Surgery Branch, National Cancer Institute with approved protocol 06-C-0014 (NCT 00242723) by National [illegible]
12. Include a statement confirming that informed consent was obtained from all subjects.	Informed consent was obtained from all patients (line 395).
13. For publication of patient photos, include a statement confirming that consent to publish was obtained.	not applicable (NA)
14. Report the clinical trial registration number (at ClinicalTrials.gov or equivalent).	NA

15. For phase II and III randomized controlled trials, please refer to the CONSORT statement and submit the CONSORT checklist with your submission.

16. For tumor marker prognostic studies, we recommend that you follow the REMARK reporting guidelines.

Data deposition

Reported in section/paragraph or page #

17. Provide accession codes for deposited data. Data deposition in a public repository is mandatory for:<br>a. Protein, DNA and RNA sequences<br>b. Macromolecular structures<br>c. Crystallographic data for small molecules<br>d. Microarray data	Accession numbers for the original microarray data (accession no. GSE65291) were deposited at the NCBI Gene Expression Omnibus (http://www.ncbi.nlm.nih.gov/geo/query/acc.cgi?acc=GSE65291).

Deposition is strongly recommended for many other datasets for which structured public repositories exist; more details on our data policy are available in the Guide to Authors. We encourage the provision of other source data in supplementary information or in unstructured repositories such as Figshare and Dryad. We encourage publication of Data Descriptors (see Scientific Data) to maximize data reuse.

18. If computer code was used to generate results that are central to the paper's conclusions, include a statement in the Methods section under "**Code availability"** to indicate whether and how the code can be accessed. Include version information as necessary and any restrictions on availability.	NA
